# Hydrostatic pressure reduces the mechanosensitivity of cell migration

**DOI:** 10.1126/sciadv.aed0981

**Published:** 2026-06-26

**Authors:** Ayuba Akinpelu, Abby Weaver, Farnaz Hemmati, Ethan Coker, Farshad Amiri, Chrystalla Stylianou, Ravi S. Vaghasiya, Matthew T. Garnett, Daniel Nweze, Maria Kalli, Symone Alexander, Triantafyllos Stylianopoulos, Yizeng Li, Panagiotis Mistriotis

**Affiliations:** ^1^ Department of Chemical Engineering, Auburn University, Auburn, AL 36849, USA.; ^2^ Cancer Biophysics Laboratory, Department of Mechanical Engineering, University of Cyprus, Nicosia 2109, Cyprus.; ^3^ Department of Biomedical Engineering, Binghamton University, SUNY, Binghamton, NY 13902, USA.

## Abstract

Cells sense and respond to diverse physical cues as they migrate toward distant sites. While much is known about the roles of cellular molecules in the regulation of mechanosensitivity, our understanding of how extracellular cues influence this property remains limited. Here, we show that prolonged exposure to elevated, yet (patho)physiologically relevant, extracellular hydrostatic pressure decreases migration sensitivity to substrate stiffness, fluid viscosity, fluid forces, and hydraulic resistance. Reduced mechanosensitivity can persist for days after the high-pressure cue is removed, indicating that cells retain a memory of hydrostatic pressure. Mechanistically, high pressure down-regulates the Rho/MRTF/SRF pathway, activating a myosin II–independent mechanosensing mechanism that shifts the maximum cell speed toward stiffer substrates, as predicted mathematically and demonstrated experimentally. Stiffer substrates increase migration of preconditioned cells by strengthening focal adhesions and redistributing them to the cell periphery to support Arp2/3-dependent lamellipodia extension. Collectively, hydrostatic pressure reprograms the mechanosensing machinery to drive lasting effects on cell mechanosensitivity.

## Teaser

Pressure-induced suppression of SRF activity rewires the mechanosensing machinery, reducing cell migration mechanosensitivity.

## INTRODUCTION

In vivo, migrating cells are subjected to diverse physical cues [e.g., extracellular matrix (ECM) stiffness, extracellular fluid viscosity, confinement, solid stress, hydraulic resistance, and fluid forces], which modulate the activity, levels, or spatial organization of migration machinery components, thereby shaping the direction and efficiency of cell locomotion (*1*–*3*). Cells perceive and interpret the physical microenvironment through mechanosensors, which work cooperatively to convert mechanical signals into biochemical signaling pathways, influencing downstream processes. Plasma membrane and intracellular mechanosensors [e.g., integrins, mechanosensitive ion channels (MIC), and cytoskeletal elements] are critical for controlling the mechanosensitivity of cell migration (*2*, *4*), defined as a cell’s ability to sense mechanical cues and respond by adjusting its migration speed, persistence, or direction. To date, the extracellular regulation of cell migration mechanosensitivity remains largely unexplored.

Metastatic cancer cells are a prime example of migratory cells, navigating environments rich in physical signals during their journey from the primary tumor to distant organs (*5*). Emerging evidence suggests that metastatic cancer cells display altered mechanosensitivity relative to their nonmetastatic or nonmalignant counterparts. MDA-MB-231 breast cancer cells capable of invading the stroma have reduced adhesion strength compared to those retained within the primary tumor (*6*). In contrast to their strongly adherent counterparts, weakly adherent cells form more metastases and remain largely unresponsive to stiffness gradients that exist between the tumor microenvironment and the surrounding stroma (*6*, *7*), potentially explaining how this subpopulation migrates away from the stiffer primary tumor. Moreover, highly invasive and metastatic breast cancer cells have lower levels of the nuclear envelope protein lamin A (*8*), whose depletion suppresses migration sensitivity to stiffness (*9*). Using tumor subpopulation cell lines from the isogenic 4T1 breast cancer progression series, we identified an inverse correlation between metastatic potential and the ability of cancer cells to sense fluid forces in confined spaces, with reduced sensitivity favoring escape from high-pressure environments, similar to those in the primary tumor (*10*). Shear sensing is also reduced in fibroblast-derived cancer cells (HT-1080 fibrosarcoma cells) compared to normal fibroblasts, enabling these tumor cells to enter regions of higher shear stress, such as intravasation sites (*11*). Increasing shear sensitivity by overexpressing the MIC transient receptor potential melastatin 7 prevents HT-1080 cells from penetrating into blood vessels and forming invasive metastatic lesions in vivo (*11*).

While genetic changes could contribute to the observed differences in mechanosensitivity between metastatic cells and nonmetastatic/nonmalignant cells, we reasoned that the tumor microenvironment, given its key role in regulating cancer aggressiveness and dissemination (*12*, *13*), provides signals that alter how cells perceive physical cues. We focused on extracellular hydrostatic pressure because (i) it is consistently and uniformly elevated across a wide range of solid tumors, typically reaching values in the kilopascal range in human cancers (*14*); (ii) it has been found to scale with tumor size and to distinguish invasive from noninvasive carcinomas (*15*); and (iii) at least in some cancer types, increased pressure associates with metastasis (*16*–*18*), pointing to a possible link with invasion and metastatic spread. Using a microchannel device to tune the levels of hydrostatic pressure, we find that prolonged exposure to hydrostatic pressure of 1 kPa down-regulates the Rho GTPase (Rho)/Myocardin-Related Transcription Factor (MRTF)/Serum Response Factor (SRF) pathway, rewiring the mechanosensing machinery and thereby reducing the sensitivity of migration to diverse physical cues.

## RESULTS

### Elevated hydrostatic pressure reduces cell sensitivity to substrate stiffness

**Fig. 1.**
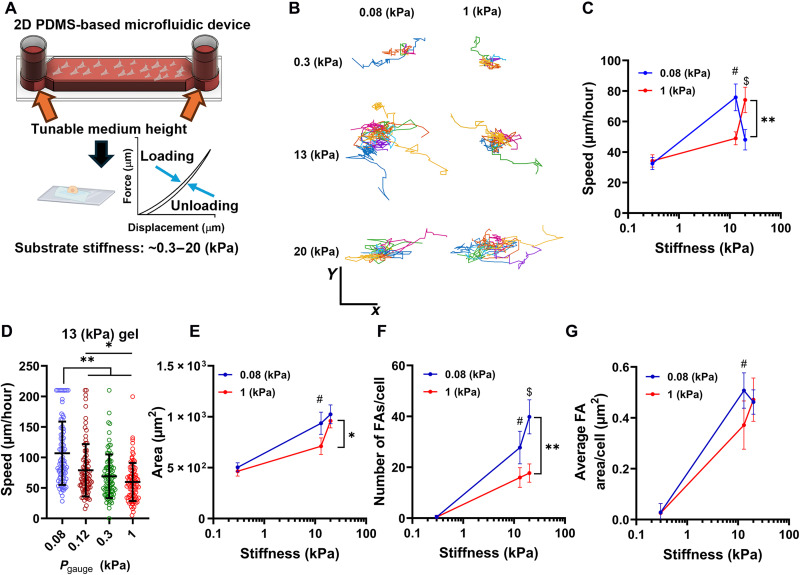
Pretreatment at high pressure triggers a rightward shift in the optimal stiffness for cell migration. (**A**) Schematic of the device used to pretreat cells at varying hydrostatic pressures, followed by exposure to different stiffness substrates. The Young’s modulus was calculated using the slope of the unloading curve. (**B** and **C**) Ten representative trajectories (B) and speed (C) of pretreated (0.08 or 1 kPa) cells on different stiffness substrates (*n* = 89 per condition, *N* = 3). **^,$,#^*P* < 0.01 ($: 1-kPa cells on 13-kPa versus 1-kPa cells on 20 kPa; #: 0.08-kPa cells on 13-kPa versus 1-kPa cells on 13 kPa). Scale bar, 50 μm. (**D**) Speed of pretreated (0.08, 0.12, 0.3, and 1 kPa) cells on 13-kPa substrates (*n* = 90 per condition, *N* = 3). *P < 0.05 and **P < 0.01. (**E**) Projected area of pretreated (0.08 or 1 kPa) cells on different stiffness substrates (*n* = 105, *N* = 3). *P < 0.05 and ^#^*P* < 0.01 (#: 0.08-kPa cells on 13-kPa versus 1-kPa cells on 13 kPa). (**F** and **G**) Focal adhesion (FA) number (F) and FA average area (G) in pretreated (0.08 or 1 kPa) cells on different stiffness substrates (*n* = 51 per condition, *N* = 3). ^#^*P* < 0.05 and **^,$^*P* < 0.01, (#: 0.08-kPa cells on 13-kPa versus 1-kPa cells on 13 kPa; $: 0.08-kPa cells on 13-kPa versus 0.08-kPa cells on 20 kPa). All data were generated using MDA-MB-231 cells. Pretreatments: 4 days. Statistics: two-way analysis of variance (ANOVA) followed by Tukey’s test [(C) and (E) to (G)] and Kruskal-Wallis test followed by Dunn’s test (D). Values represent mean ± 95% confidence interval [CI; (C) and (E) to (G)] or mean ± SD (D).

To expose migratory cells to varying hydrostatic pressures, we cultured them within a wide polydimethylsiloxane (PDMS)–based, collagen I–coated channel [*W*(idth) × *H*(eight) × *L*(ength) = 1 cm × 60 μm × 4 cm] flanked by two open-to-atmosphere reservoirs, which allowed adjustment of the culture medium height and thus precise control of the applied hydrostatic pressure (Fig. 1A). We applied prescribed gauge pressures to cells, ranging from 0.08 to 1 kPa, which were (patho)physiologically relevant (*14*, *15*) and several orders of magnitude lower than those reported to compromise cell survival (*19*, *20*). We also confirmed that MDA-MB-231 breast cancer cells maintained ~100% viability after 4 days of culture under 0.08 or 1 kPa (fig. S1, A and B). Following preconditioning for 1 or 4 days at different pressures, MDA-MB-231 cells were transferred to a low-pressure environment (~0.05 kPa) on collagen I–coated substrates with stiffness ranging from 0.3 to 20 kPa, spanning the physiological stiffness of most human tissues (Fig. 1A and fig. S1C) (*21*, *22*). Regardless of the pretreatment duration, cells preexposed to low pressure (0.08 kPa)—hereafter referred to as low-pressure (LP) cells—displayed a biphasic migration response to increasing substrate stiffness with maximum speed occurring at intermediate stiffness (~13 kPa; Fig. 1, B and C, and fig. S1D). This migration behavior closely mimicked the stiffness sensitivity of MDA-MB-231 cells directly from culture flasks (fig. S1E), which are typically maintained at similarly low hydrostatic pressure. Cells preexposed for 4 days to pressures exceeding 0.08 kPa showed reduced cell migration on 13-kPa substrates, with the lowest speeds observed at pressures ≥ 0.3 kPa (Fig. 1D). Cells preconditioned for 4 days at high pressure (1 kPa)—hereafter referred to as high-pressure (HP) cells—required stiffer (20 kPa) substrates to achieve peak migration speed (Fig. 1, B and C), suggesting a reduction in their ability to sense changes in matrix rigidity. These data also held true for HT-1080 fibrosarcoma cells (fig. S1F). Of note, this rightward shift in the optimal stiffness for migration was not observed in cells pretreated at 1 kPa for only 1 day (fig. S1D), indicating that prolonged exposure to elevated pressure is required to induce this effect.

The reduced sensitivity of HP cells to substrate stiffness was further supported by quantitative analysis of morphological parameters known to associate with migration speed, such as cell area and elongation, which was measured via aspect ratio (*23*, *24*). Spreading and elongation increased with substrate stiffness in LP MDA-MB-231 cells, peaking at intermediate levels and showing only minor, nonsignificant changes thereafter (Fig. 1E and fig. S1G). These observations were consistent with prior work on flask-grown MDA-MB-231 cells (*25*). HP cells were smaller and less elongated than LP cells on 13-kPa substrates, but their area and aspect ratio increased on 20-kPa substrates, reaching values comparable to those of LP cells (Fig. 1E and fig. S1G). Because changes in substrate stiffness can be sensed through adhesion molecules (*26*), we quantified focal adhesions (FAs), which link the cytoskeleton to the ECM, influencing cell migration and morphology (*27*–*29*). Specifically, we performed immunofluorescence and quantitative analysis of paxillin, an adapter protein that is a key part of the newly formed adhesions and serves as a FA marker (*30*, *31*). FA number increased monotonically with stiffness in LP MDA-MB-231 cells; however, this increase was less pronounced in HP cells, which had fewer FAs than LP cells on both 13- and 20-kPa surfaces (Fig. 1F). Stiffness-induced changes in FA area resembled those in cell area and aspect ratio; on 13-kPa substrates, HP cells had smaller FAs than LP cells, whereas on 20-kPa surfaces, this difference disappeared mainly because FA area increased in HP cells (Fig. 1G). A reduction in FA number and size on 13-kPa substrates was similarly observed in HP HT-1080 cells stably expressing paxillin–green fluorescent protein (GFP) (fig. S1, H and I).

Together, these findings indicate that on softer (13 kPa) substrates, HP cells display fewer and smaller FAs, reduced area and elongation, and decreased migration compared with LP cells. A further increase in substrate stiffness promotes FA maturation, spreading, elongation, and faster motility in HP cells, but not in LP cells, whose migration decreases.

While the high oxygen permeability of native PDMS allows adequate oxygen supply to cells, surface modifications (e.g., oxygen plasma treatment) or protein adsorption can reduce oxygen transfer through this polymer (*32*, *33*). To ensure that the observed effects of pressure were not due to PDMS-mediated hypoxia, we conducted two distinct experiments. First, we reasoned that if cells in our microfluidic chamber were truly hypoxic, culturing them in a hypoxic environment [1% (v/v) O_2_] would not significantly alter the green fluorescence intensity (GFI) of the cell-permeable oxygen indicator Image-iT, which is expected to increase if at any time intracellular oxygen levels drop below 5%. Instead, we observed that hypoxic conditions markedly increased the Image-iT GFI by ~4 times (fig. S1J). Second, we investigated the effects of hypoxia on cellular mechanosensitivity. LP cells pretreated at 1% (v/v) O_2_ for 4 days, followed by cell exposure to varying levels of substrate stiffness, resulted in migration behavior nearly identical to that of LP cells preexposed to normoxia [21% (v/v) O_2_; fig. S1K]. Furthermore, we studied whether changes in nutrient availability within the microfluidic channel would influence the stiffness sensitivity of cells. Varying the concentration of fetal bovine serum (FBS) during the pretreatment period had no effect on cell migration responses to substrates of different rigidities (fig. S1L). These findings strongly suggest that oxygen and nutrient availability are unlikely to contribute to the observed differences in stiffness sensitivity between the LP and HP cells.

### Elevated pressure broadly affects the mechanosensitivity of migratory cells

**Fig. 2.**
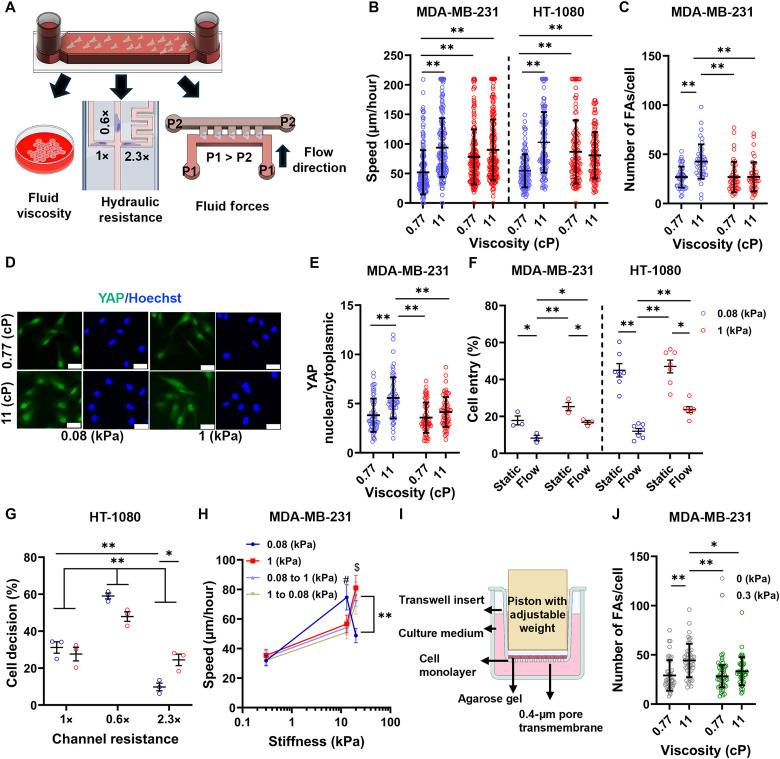
Pretreatment at high hydrostatic pressure reduces mechanosensitivity. (**A**) Schematic of pressure-preconditioned cells exposed to the indicated physical cues. (**B**) Speed of pretreated (0.08 or 1 kPa) cells on glass slides in media of different viscosities (*n* ≥ 120 per condition, *N* ≥ 4). **P < 0.01. (**C** to **E**) FA number (C), representative epifluorescence images of YAP (D), and YAP nuclear-to-cytoplasmic ratio (E) in pretreated (0.08 or 1 kPa) cells on glass slides in media of different viscosities [(C): *n* = 45 per condition, *N* = 3; E: *n* = 60 per condition, *N* = 3]. **P < 0.01. Scale bars, 20 μm. (**F**) Percentage of pretreated (0.08 or 1 kPa) cells that enter confined microchannels under static or flow (Δ*P* = –0.16 kPa) conditions (≥100 cells analyzed/experiment, *N* ≥ 3). *P < 0.05 and **P < 0.01. (**G**) Distribution pattern of pretreated (0.08 or 1 kPa) cells in microchannels of different hydraulic resistances (≥32 cells analyzed per experiment, *N* = 3). *P < 0.05 and **P < 0.01. (**H**) Cell speed on substrates of varying stiffness after 8-day pretreatment at 0.08 or 1 kPa or after 4-day at 0.08 or 1 kPa, followed by 4 days under the alternate pressure (*n* = 99 per condition, *N* = 3). **^, $, #^*P* < 0.01 ($: 1, 0.08 to 1, and 1 to 0.08 kPa cells on 13 kPa versus same conditions on 20 kPa; #: 0.08 kPa on 13 kPa versus all other conditions on 13 kPa). (**I**) Schematic of the compression device. (**J**) FA number in precompressed (~0 or 0.3 kPa) cells on glass slides in media of different viscosities (*n* = 45 per condition, *N* = 3). *P < 0.05 and **P < 0.01. Pretreatments: 4 days [(B) to (H)]; 2 days (J). 0.08 kPa (blue); 1 kPa (red). Statistics: two-way ANOVA followed by Tukey’s test. Values: mean ± SD [(B), (C), (E), and (J)], mean ± SEM [(F) and (G)], or mean ± 95% CI (H).

Next, we investigated whether pressure pretreatment altered migration responses to physical cues other than stiffness, such as fluid viscosity, fluid forces, and hydraulic resistance (Fig. 2A and movies S1 to S4). Prior studies have demonstrated that cells increase their migration speed in culture medium whose viscosity is experimentally raised above that of water to approximate the viscosities of bodily fluids (*34*–*37*). Consistent with these findings, MDA-MB-231 and HT-1080 cells directly from culture flasks or preexposed to 0.08 kPa for 4 days migrated faster in high-viscosity medium (11 cP) compared to medium with a viscosity similar to that of water (0.77 cP; Fig. 2B and fig. S2A). Viscosity-induced increases in migration speed were nearly abolished in HP cells (Fig. 2B and fig. S2B), and this result was independent from substrate stiffness, as HP cells showed markedly diminished responses to elevated viscosity on both rigid glass slides (Fig. 2B) and compliant 0.3-kPa substrates (fig. S2B). Of note, HP cells migrated faster on glass in low-viscosity medium than LP cells under the same viscosity conditions (Fig. 2B), consistent with the higher HP cell speed on stiff substrates (Fig. 1, B and C, and fig. S1F). To further validate the reduced ability of HP cells to respond to fluid viscosity, we measured viscosity-induced FA formation that supports cell spreading and faster migration in viscous fluids (*34*–*36*). We also evaluated YAP nuclear translocation, a key mediator of mechanical memory to viscosity (*35*). In line with our speed measurements, pretreatment at high hydrostatic pressure abrogated the viscosity-induced increases in FA number, cell area, and the nuclear-to-cytoplasmic ratio of YAP (Fig. 2, C to E, and fig. S2, C and D).

To study fluid force–dependent cell responses, we used a previously optimized microfluidic assay (*10*), in which cells entering tightly confined microchannels (*W* × *H* × *L* = 10 μm by 3 μm by 200 μm) coated with collagen I were subjected to fluid forces generated by a pressure differential (Δ*P* = *P*_2_ – *P*_1_) of −0.16 kPa across these microchannels (Fig. 2A). We have recently shown that fluid forces counterintuitively inhibit entry into microchannels of flask-grown HT-1080 and MDA-MB-231 cells, nearly halting confined migration in the direction of flow (*10*). In agreement with this, LP and HP cells exhibited reduced channel entry in the presence of flow relative to static conditions (Fig. 2F). However, this reduction was less pronounced in HP cells, as they entered confined microchannels in greater proportions compared with LP cells (Fig. 2F and movies S1 and S2), suggesting that HP cells are less sensitive to fluid forces. Furthermore, we investigated the directional choices made by cells when navigating confining paths with varying hydraulic resistances. To investigate this, we used a microfluidic device containing an array of trifurcating, Ψ-like, collagen I–coated microchannels (*38*) with a constant cross-sectional area (*W* × *H* = 10 μm by 3 μm) but different lengths (straight: 180 μm; left: 300 μm; right: 700 μm; Fig. 2A). Because hydraulic resistance is proportional to channel length (*38*), cells at the intersection were exposed to relative hydraulic resistances of 0.6×, 1×, and 2.3× for the straight, left, and right branches, respectively. Cells directly from flasks preferentially chose the straight branch (lowest resistance channel), followed by the left branch (intermediate resistance channel), and rarely entered the right branch (highest resistance channel) (fig. S2E). Similar to flask-grown cells, LP and HP cells preferentially entered the straight branch (Fig. 2G and movies S3 and S4). Unlike LP and flask-grown cells, HP cells failed to distinguish between intermediate and high hydraulic resistance channels, entering the right and left branches with equal probability (Fig. 2G and movies S3 and S4). Collectively, these findings reveal that long-term exposure to elevated hydrostatic pressure reduces the mechanosensitivity of cell migration. MDA-MB-231 cells retained reduced mechanosensitivity for at least 4 days following discontinuation of the high-pressure stimulus, as shown in experiments in which cells were cultured at 0.08 or 1 kPa for 4 days, followed by exposure to a high- (1 kPa) or low- (0.08 kPa) pressure environment for an additional 4 days (Fig. 2H and fig. S2, F to I). These data indicate that MDA-MB-231 cells develop long-term memory of hydrostatic pressure. The duration of this memory varies between cell types, as HT-1080 cells regained mechanosensitivity faster, within 4 days after the pressure signal is removed (fig. S2, H and I).

Because hydrostatic pressure is the pressure exerted on a cross-sectional area due to the mass of fluid above it, we investigated whether compressive loading produces effects comparable to those triggered by hydrostatic pressure–induced stress. To this end, we applied dorsoventral compression to cells using a transmembrane pressure device (Fig. 2I) (*39*, *40*). In this setup, MDA-MB-231 cells were covered with an agarose gel, on top of which a weighted piston was placed to apply a pathophysiologically relevant compressive stress of 0.3 kPa for 2 days, as higher stresses or longer durations compromised cell viability (*39*, *41*). Control cells were covered with agarose gel only (~0 Pa). In agreement with our observations from hydrostatic pressure experiments, cell preconditioning at 0.3 kPa eliminated the viscosity-induced increases in FA formation and YAP nuclear translocation (Fig. 2J and fig. S3, A and B), suggesting that prolonged exposure to elevated compressive stress diminishes cell mechanosensing.

### HP cells use a myosin II–independent mechanosensing mechanism

**Fig. 3.**
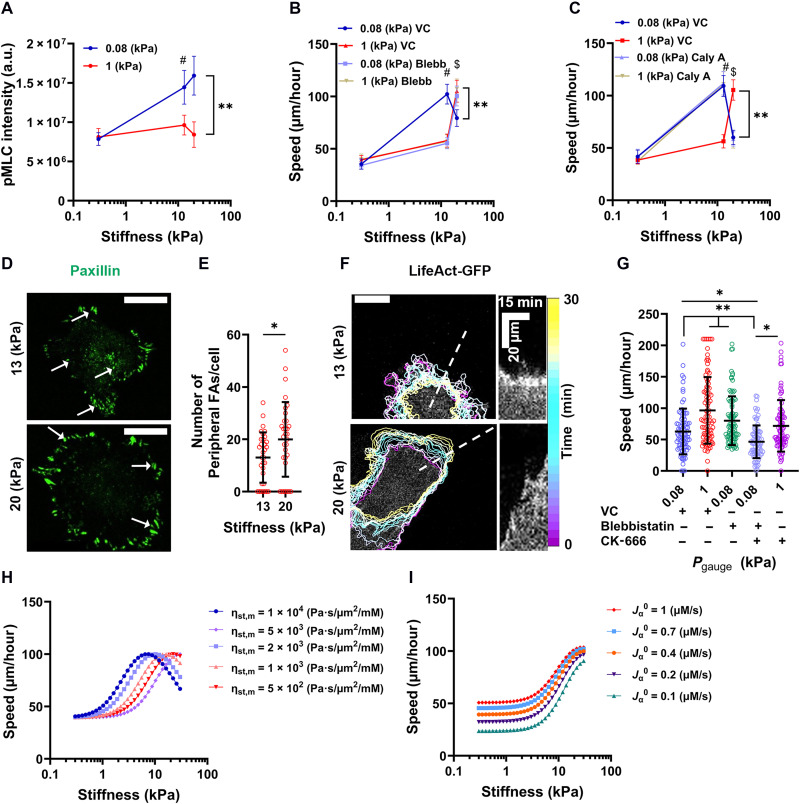
Actin polymerization drives stiffness-induced, myosin II–independent migration. (**A**) pMLC levels in pretreated (0.08 or 1 kPa) cells on different stiffness substrates (*n* = 80 per condition, *N* = 4). **^,#^*P* < 0.01 (#: 0.08-kPa cells on 13-kPa versus 1-kPa cells on 13 kPa). a.u., arbitrary units. (**B** and **C**) Speed of preconditioned (0.08 or 1 kPa) cells treated with vehicle control (VC), blebbistatin (Blebb), or calyculin A (Caly A) on different stiffness substrates (*n* = 90 per condition, *N* = 3). **^,$,#^*P* < 0.01 (B: $: 1-kPa VC, 0.08-kPa Blebb, and 1-kPa Blebb on 13-kPa versus same conditions on 20 kPa; #: 0.08-kPa VC on 13-kPa versus all other conditions on 13 kPa; C: $: 1-kPa VC on 13-kPa versus 1-kPa VC on 20 kPa; #: 1-kPa VC on 13-kPa versus all other conditions on 13 kPa). (**D** and **E**) Representative confocal images (D) and peripheral FA number (E) in pretreated (1 kPa) cells on different stiffness substrates (*n* = 30 per condition, *N* = 3). Arrows show FAs. *P < 0.05. Scale bars, 10 μm. (**F**) Contours (scale bar, 35 μm) and kymographs of pretreated (1 kPa), LifeAct-GFP–labeled cells on different stiffness substrates. (**G**) Speed of preconditioned (0.08 or 1 kPa) cells treated with VC, Blebb, VC and CK-666, or Blebb and CK-666 on 20-kPa substrates (*n* = 90 per condition, *N* = 3). *P < 0.05 and **P < 0.01. (**H**) Cell speed versus substrate stiffness at varying $\eta_{\text{st},m}$. (**I**) Cell speed versus substrate stiffness for $\eta_{\text{st},m}=500\;\text{Pa}\cdot s\cdot {\mathrm{\mu m}}^{-2}\cdot {\text{mM}}^{-1}$ and at varying *J*_α_^0^. Data in (A) to (G) were generated using MDA-MB-231 cells. Pretreatments: 4 days. Statistics: two-way ANOVA followed by Tukey’s test [(A) to (C)], Mann-Whitney test (E), and Kruskal-Wallis test followed by Dunn’s test (G). Values: mean ± 95% CI [(A) to (C)] and mean ± SD [(E) and (G)].

We next sought to identify the mechanisms underlying the reduced sensitivity of HP cells to substrate stiffness. The smaller FAs in HP cells on 13-kPa substrates (Fig. 1G and fig. S1I), compared with LP cells, suggest reduced myosin II–mediated contractile forces that promote FA maturation (*30*, *42*, *43*). Consistent with this, immunofluorescence analysis of phosphorylated myosin light chain (pMLC) revealed that the stiffness-induced increase in myosin II activation, observed in LP cells, was completely abolished in HP cells (Fig. 3A). To test how down-regulation of myosin II activity affected stiffness sensitivity, we treated LP and HP cells with the myosin II adenosine triphosphatase cycle inhibitor blebbistatin (25 μM). Blebbistatin triggered a rightward shift in the optimal stiffness for LP cell migration, mirroring the effects of high-pressure pretreatment while leaving the stiffness sensitivity of HP cells unchanged (Fig. 3B). Similar results were obtained with 10 μM ROCK inhibitor Y-27632 (fig. S4A), which blocks MLC phosphorylation (*44*). Conversely, treatment with the serine/threonine phosphatase inhibitor calyculin A (0.1 nM), which enhances contractility by increasing MLC phosphorylation (*45*), restored stiffness sensitivity in HP cells without altering that of LP cells (Fig. 3C). These data indicate that HP cells use a myosin II–independent mechanosensing mechanism, which underlies the rightward shift in the optimal stiffness for cell migration.

While HP cells exhibited a similar overall number of FAs on 13- and 20-kPa substrates (Fig. 1F), radial quantification within 5 μm of the cell edge revealed increased peripheral FA formation on 20-kPa relative to 13-kPa substrates (Fig. 3, D and E). FAs at the cell leading edge anchor filamentous actin (F-actin) to the substrate, allowing the growing actin filaments to push against the plasma membrane and drive lamellipodia extension and migration (*46*, *47*). By monitoring the lamellipodial growth using LifeAct-GFP–labeled cells, we found that stiffer (20 kPa) substrates promoted stable and continuous protrusive activity in HP cells, leading to pronounced extension of the cell leading edge, as evident from the kymograph (Fig. 3F and fig. S4B). By comparison, softer (13 kPa) substrates induced unsteady protrusions, inhibiting lamellipodia extension and limiting HP cell spreading and locomotion (Fig. 3F and fig. S4B). Cell treatment with CK-666 (100 μM), which specifically inhibits Arp2/3 (*48*) [a protein complex that localizes in lamellipodia to nucleate branched actin filaments and drive lamellipodia extension (*49*–*51*)] markedly suppressed the migration speed of HP cells or blebbistatin-treated cells on 20-kPa substrates (Fig. 3G). These data suggest that Arp2/3-dependent actin polymerization at lamellipodia is a key driver of stiffness-induced, myosin II–independent migration.

To quantitatively understand how changes in actomyosin engagement and actin polymerization affect stiffness-induced migration, we developed a simplified one-dimensional (1D) theoretical model linking actin polymerization to FAs, with FA strength depending on substrate stiffness and actomyosin engagement. The model includes the interconversion between F-actin and globular actin (G-actin) through polymerization and depolymerization. The F-actin network is mechanically connected to FAs to provide the driving force for cell migration. The FA strength determines how much force is transmitted to the actin network and is modeled as $\eta_{\text{st}}=\eta_{\text{st},0}+\eta_{\text{st},m}{\left(k_{\text{st}}/k_{\text{st},0}\right)}^{2}$, where $\eta_{\text{st},0}$ is the baseline FA strength, $k_{\text{st}}$ is the stiffness of the substrate, $k_{\text{st},0}$ is a stiffness normalization factor, and $\eta_{\text{st},m}$ is the coefficient of FA influenced by actomyosin network engagement. If actomyosin network is less engaged in establishing FAs, as in the case of HP cells or blebbistatin-treated cells, $\eta_{\text{st},m}$ decreases, leading to low-strength adhesions and reduced force transmission at a fixed substrate stiffness. In addition, lower substrate stiffness diminishes FA strength and force transmission at a given $\eta_{\text{st},m}$. The details of the model can be found in Materials and Methods.

The model predicts that decreasing $\eta_{\text{st},m}$ shifts the maximum cell speed toward higher substrate stiffness (Fig. 3H), consistent with the experimental observations (Figs. 1C and 3B and fig. S1F). The model also quantifies how the rate of actin polymerization influence speed in cells with low actomyosin engagement ($\eta_{\text{st},m}=500\;\text{Pa}\cdot s/{\mathrm{\mu m}}^{2}/\text{mM}$). In such cells, suppressing actin polymerization by reducing the coefficient of actin polymerization (*J*_α_^0^) decreases cell speed for all stiffness range (Fig. 3I), in line with our experimental results shown in Fig. 3G. Collectively, our findings indicate that in HP cells, 20-kPa surfaces redistribute and strengthen FAs, enabling the transmission of greater forces between the substrate and cytoskeleton and stabilizing lamellipodial protrusions, thereby increasing migration compared to softer substrates.

### Down-regulation of the Rho/MRTF/SRF pathway mediates the effects of hydrostatic pressure on the mechanosensitivity of cell migration

The requirement for prolonged cell culture under elevated pressure to reduce cell migration mechanosensitivity implies that transcriptional changes are involved. It is well established that the Rho/MRTF/SRF signaling pathway promotes the expression of genes critical for mediating cell mechanosensing and mechanoresponses, including those involved in cell-ECM adhesion and activation of actomyosin contractility (*52*–*54*). Our results indicate that HP cells display reduced myosin II activation and FA formation or maturation in response to physical stimuli (Figs. 1, F and G, 2C, and 3A and fig. S1, H and I, and S2C) and exhibit mechanosensitivity nearly identical to that of LP cells treated with contractility inhibitors (Fig. 3B and fig. S4A). These data prompted us to hypothesize that elevated hydrostatic pressure suppresses the Rho/MRTF/SRF signaling pathway, leading to changes in the mechanosensing machinery that underlie the reduced mechanosensitivity of HP cells.

**Fig. 4.**
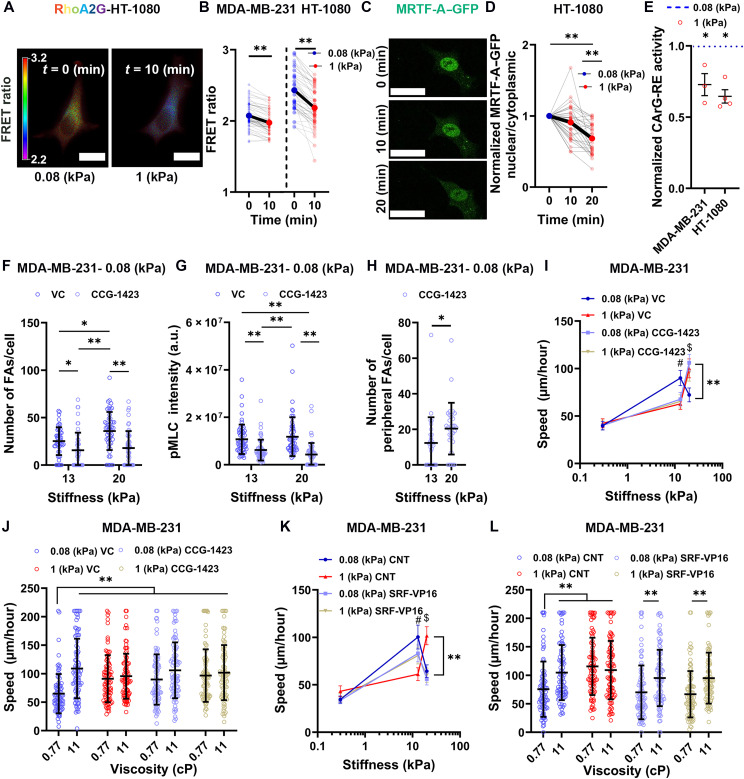
Hydrostatic pressure down-regulates the Rho/MRTF/SRF pathway. (**A** and **B**) RhoA FRET ratio images (A) and quantification (B) at 0.08 versus 1 kPa (*n* ≥ 51 per condition, *N* = 3). **P < 0.01. Scale bars, 10 μm. (**C** and **D**) MRTF-A–GFP confocal images (C) and nuclear-to-cytoplasmic ratio quantification (D) at 0.08 versus 1 kPa (*n* = 30, *N* = 3). **P < 0.01. Scale bars, 35 μm. (**E**) Normalized CArG-RE activity after 4 days under 0.08 or 1 kPa (10 images per experiment, *N* ≥ 3). *P < 0.05. (**F** and **G**) FA number [(F); *n* = 51 per condition, *N* = 3] and pMLC levels [(G); *n* = 60 per condition, *N* = 3] in pretreated (0.08 kPa with VC or CCG-1423) cells on different stiffness substrates. *P < 0.05 and **P < 0.01. (**H**) Peripheral FA number in pretreated (0.08 kPa with CCG-1423) cells on different stiffness substrates (*n* = 30 per condition, *N* = 3). *P < 0.05. (**I** and **J**) Speed of pretreated (0.08 or 1 kPa with VC or CCG-1423) cells on different stiffness substrates (I) or in media of different viscosities [(J); *n* = 90 per condition, *N* = 3]. **^,$,#^*P* < 0.01 ($: 1-kPa VC/CCG-1423 and 0.08-kPa CCG-1423 on 13 kPa versus same conditions on 20 kPa; #: 0.08-kPa VC on 13 kPa versus all other conditions on 13 kPa). (**K** and **L**) Speed of pretreated (0.08 or 1 kPa) and subsequently transduced [with a control vector (CNT) or SRF-VP16] cells on different stiffness substrates (K) or in media of different viscosities [(L); *n* = 90 per condition; *N* = 3]. **^,$,#^*P* < 0.01 ($: 1-kPa CNT on 13 kPa versus 1-kPa CNT on 20 kPa; #: 1-kPa CNT on 13 kPa versus all other conditions on 13 kPa). Pretreatments [(F) to (L)]: 4 days. Statistics: paired *t* test (B), Friedman tests followed by Dunn’s test (D), unpaired *t* test (E), two-way ANOVA followed by Tukey’s test after log transformation (F), two-way ANOVA followed by Tukey’s test [(G) and (I) to (L)], and Mann-Whitney test (H). Thick black lines [(B) and (D)] represent averages. Values: mean ± 95% CI [(I) and (K)], mean ± SD [(F) to (H), (J), and (L)], mean ± SEM (E), and symbols and lines [(B) and (D)].

To test our hypothesis, we first quantified RhoA activity using an optimized Förster resonance energy transfer (FRET)–based RhoA activity biosensor (*42*, *55*–*57*). This biosensor detects RhoA activation through elevated FRET signals, resulting from guanosine 5′-triphosphate (GTP)–loaded RhoA binding to the rhotekin Rho domain, which brings the mTFP-1 (cyan) donor into proximity with the yellow fluorescent protein (YFP) acceptor. We confirmed that the RhoA activity biosensor worked as expected by demonstrating that treatment of HT-1080 cells with lysophosphatidic acid (LPA; 50 μM) markedly increased FRET emission intensity ratio (fig. S5A). To assess the effects of elevated pressure on RhoA activity, we seeded MDA-MB-231 and HT-1080 cells expressing the RhoA biosensor on collagen I–coated substrates and cultured them under 0.08 kPa (fig. S5B). Increasing the hydrostatic pressure to 1 kPa by raising an open-ended tube filled with culture medium to the appropriate height while sealing the tube at the opposite side to minimize fluid disturbance (fig. S5B) led to a decrease in RhoA activity (~5% for MDA-MB-231 and ~10% for HT-1080 cells) after 10 min (Fig. 4, A and B). By comparison, cells kept at 0.08 kPa during the same period showed only minor changes (≤2%) in RhoA activity (fig. S5C). RhoA activation leads to F-actin formation, enabling MRTF-A to translocate to the nucleus and bind to the transcription factor SRF, thereby inducing the expression of a subset of SRF target genes (*52*). Using the MRTF-A–GFP live-cell reporter, which showed the expected nuclear localization of MRTF-A under serum stimulation [10% (v/v)] and exportin 1 inhibition (*58*) (KPT-330; 10 μM; fig. S5D), we found that acute exposure of HT-1080 cells to 1-kPa pressure resulted in MRTF-A–GFP nuclear exit after 20, but not 10, min (Fig. 4, C and D), suggesting a delayed response that follows or coincides with the down-regulation of RhoA activity. Moreover, we treated MRTF-A–GFP–labeled HT-1080 cells with LPA, which increased RhoA activity (fig. S5A) and subsequently exposed them to a hydrostatic pressure of 1 kPa for 20 min. LPA treatment abolished hydrostatic pressure–induced MRTF-A–GFP nuclear exit (fig. S5, E and F), suggesting that RhoA activation blocks hydrostatic pressure sensing. Despite greater cell-to-cell variability, MRTF-A–GFP localization remained unchanged on average in control cells maintained at 0.08 kPa throughout the 20-min period (fig. S5, G and H). Furthermore, we used a previously optimized live-cell fluorescent reporter to quantify SRF transcriptional activity (*59*, *60*), also referred to as CArG–Response Element (CArG-RE) activity. Cells cultured under 1-kPa pressure for 4 days displayed reduced CArG-RE activity compared to those maintained at 0.08 kPa (Fig. 4E). Four-day pretreatment with CCG-1423 (10 μM), which inhibits MRTF-A/B binding to importin α/β_1_, preventing its nuclear import and thus suppressing SRF transcriptional signaling (fig. S5I) (*61*, *62*), led to decreased FA number and pMLC levels in LP cells on both 13- and 20-kPa substrates (Fig. 4, F and G), mimicking the effects of high-pressure pretreatment (Fig. 1F and 3A and fig. S1H). Moreover, similar to HP cells (Fig. 3, D and E), CCG-1423–pretreated cells showed enhanced peripheral localization of FAs on 20-kPa relative to 13-kPa substrates (Fig. 4H), suggesting that inhibition of MRTF/SRF signaling, similar to high-pressure pretreatment, increases the stiffness threshold needed to stabilize protrusions. Furthermore, cell preexposure to CCG-1423 or SRF knockdown (fig. S6A) reduced the migration sensitivity of LP cells to stiffness, fluid viscosity, and fluid forces (Fig. 4, I and J, and fig. S6, B to D), rendering their mechanical behavior similar to that of HP cells (Figs. 1C and 2, B and F, and fig. S1F). We also examined whether reactivating the Rho/MRTF/SRF pathway after high-pressure pretreatment was sufficient to rescue the diminished mechanosensitivity of preconditioned cells. Ectopic expression of a constitutively active SRF (SRF-VP16), which activates SRF-dependent gene expression independently of MRTF-A/B (*63*), restored the migration sensitivity of HP cells to stiffness, fluid viscosity, and fluid forces without altering the mechanosensitivity of LP cells (Fig. 4, K and L, and fig. S6E). Moreover, doxycycline-induced expression of constitutively active RhoA mutant [enhanced GFP (eGFP)–RhoA–Gln63→Leu(Q63L)] after the 4-day pretreatment period shifted the optimal stiffness for HP cell migration toward lower values (fig. S6F). Although LP cells and RhoA mutant–expressing cells reached their maximum migration speed on substrates of intermediate stiffness, the latter exhibited lower overall speeds (fig. S6F), consistent with the known inhibitory effects of constitutively active RhoA on cell motility (*42*, *64*, *65*). Together, these data suggest that prolonged exposure to elevated hydrostatic pressure down-regulates the Rho/MRTF/SRF pathway, limiting myosin II activation and FA formation/maturation, consequently impairing the mechanosensitivity of cell migration.

## DISCUSSION

Migrating cells actively probe their surrounding matrix, translating physical cues into biochemical signals, which allows them to adapt and navigate diverse mechanical environments. Here, we show that prolonged exposure to elevated extracellular hydrostatic pressure reduces the ability of single migratory cells to sense substrate rigidity, fluid forces, fluid viscosity, and hydraulic resistance, thereby influencing how cells adjust their morphology and migration in response to their physical microenvironment. This reduced mechanoperception persists for days after withdrawal of the hydrostatic pressure stimulus, with the strength of this memory varying between HT-1080 (<4 days) and MDA-MB-231 (>4 days) cells, presumably because the faster proliferation rate of the former (doubling time ~24 hours compared to ~36 hours for MDA-MB-231 cells) accelerates the dilution of pressure-induced memory (*66*). Our data indicate that high hydrostatic pressure reduces the Rho/MRTF/SRF pathway activity, down-regulating migration machinery components crucial for controlling the mechanosensitivity of cell migration.

SRF is a ubiquitous transcription factor that binds to the palindromic CC(A/T)_6_GG DNA sequence, known as the CArG box. SRF activity is regulated by cofactors, such as MRTFs (MRTF-A/B), which translocate to the nucleus in response to actin polymerization (*52*). We find that hydrostatic pressure of 1 kPa promotes MRTF-A nuclear export and suppresses SRF transcriptional activity, suggesting reduced expression of MRTF/SRF target genes involved in mechanosensing and mechanoresponses, including those regulating cell-ECM adhesion (e.g., ITGB1 and FA proteins) and actomyosin contractility (e.g., MYH9 and myosin light chain kinase) (*52*–*54*). Consistent with this, HP cells display impaired FA formation/maturation and myosin II activation in response to matrix rigidity and reduced mechanosensitivity. These pressure-dependent effects are mirrored by pretreatment with the specific MRTF inhibitor CCG-1423. Moreover, SRF knockdown diminishes the mechanosensitivity of LP cells, whereas constitutively active SRF restores that of HP cells. These data indicate that pressure-induced rewiring of the migration-associated mechanosensing machinery is mediated mainly through inhibition of MRTF/SRF signaling. However, we cannot exclude contributions from additional SRF-independent mechanisms, as MRTFs also cooperate with other transcriptional regulators of migration, such as signal transducer and activator of transcription 3 (STAT3) and YAP (*67*).

While the MRTF/SRF signaling pathway is generally viewed as a driver of migration and invasion, as demonstrated using diverse in vitro models, including wound-healing, Matrigel, and organotypic invasion assays (*61*, *68*–*70*), we find that the effects of MRTF/SRF are strongly shaped by physical cues of the cellular microenvironment. In MDA-MB-231 cells that express high levels of SRF (*71*), the MRTF/SRF pathway promotes cell motility on intermediate-stiffness substrates but suppresses it on stiffer ones. In addition, MRTF/SRF signaling is required for the viscosity-induced enhancement of cell motility and inhibits confined migration in the direction of flow. These findings underscore the key role of this pathway in mediating diverse migration mechanoresponses.

MRTF-dependent SRF transcriptional activation is regulated by Rho GTPases, including RhoA (*52*, *72*). In line with our data demonstrating reduced MRTF/SRF signaling under elevated hydrostatic pressure, we find a similar suppression of RhoA activity that precedes or coincides with MRTF-A nuclear export, while ectopic expression of constitutively active RhoA restores the stiffness sensitivity of HP cells. The mechanisms by which hydrostatic pressure modulates RhoA activity remain unclear. Cells maintain a positive hydrostatic pressure relative to their surrounding environment and are able to tune their intracellular pressure through diverse mechanisms, including the Rho/ROCK pathway (*56*, *73*–*77*). An acute increase in extracellular hydrostatic pressure can reduce the transmembrane Δ*P* (*P*_in_ – *P*_out_) especially if this pressure increase is not immediately transmitted across the membrane. According to the law of Laplace, a drop in transmembrane Δ*P* may lead to a reduction in effective surface tension, which reflects the combined contribution of membrane and cortical tension (*78*). Lower membrane tension suppresses MIC-mediated calcium influx, leading to reduced RhoA activity (*78*). Down-regulation of the Rho/ROCK pathway could lower intracellular pressure (*56*, *73*–*77*), thereby exacerbating the decrease in transmembrane Δ*P* and potentially further diminishing RhoA activity. Low Rho/ROCK activity could also reduce cortical tension and increase membrane tension, augmenting MIC activity (*38*, *78*). Furthermore, increased hydrostatic pressure could directly or indirectly affect microtubules whose stability regulates RhoA activity (*79*–*81*). Future work will be needed to disentangle these mechanisms and determine whether hydrostatic pressure acts on RhoA primarily by decreasing transmembrane Δ*P*, and therefore membrane tension, by driving cytoskeletal remodeling, or through a combination of both mechanisms.

LP cells, which are characterized by active Rho/MRTF/SRF signaling and a functional migration machinery, exhibit a biphasic relationship between migration speed and ECM stiffness with a maximum migration speed on 13-kPa substrates, consistent with observations in numerous and diverse cell types typically cultured under low hydrostatic pressure conditions (*24*, *82*–*85*). Pretreatment under elevated pressure reprograms the stiffness-sensing machinery by activating a myosin II–independent mechanism that shifts the optimal stiffness for migration toward higher values (~20 kPa), as predicted mathematically and demonstrated experimentally. Although HP cells display a similar number of FAs on 20- and 13-kPa substrates, the stiffer surface increases the strength of these FAs and induces their assembly preferentially at the cell edge, a process previously shown to require myosin II–independent forces in the lamellipodium (*86*). In turn, these peripheral FAs stabilize the myosin II–independent protrusions, facilitating their extension and driving spreading and faster migration on 20-kPa relative to 13-kPa substrates. While myosin motors are considered the force generators in the classical motor-clutch stochastic model, which is widely used to provide mechanistic understanding of cell sensitivity to stiffness (*24*, *87*), other studies have shown that actin polymerization alone can generate forces within the lamellipodium and drive stiffness-dependent, myosin II–independent traction (*86*, *88*). Inhibition of the Arp2/3 complex, which localizes to lamellipodia to nucleate branched actin filaments and drive lamellipodia extension (*49*–*51*), reduces the migration speed of HP cells and myosin II–inhibited LP cells on 20-kPa substrates, indicating the key role for Arp2/3-mediated actin polymerization in stiffness-dependent, myosin II–independent migration. However, other F-actin nucleators (e.g., formins) may also contribute to this process.

In addition to regulating stiffness sensing, preexposure to high hydrostatic pressure diminishes the capacity of migratory cells to perceive extracellular fluid viscosity, fluid forces, and hydraulic resistance. Previous studies have demonstrated that inhibiting myosin II activity or interfering with cell-ECM adhesion attenuates migration responses to these physical cues (*10*, *35*, *38*, *89*). These published data provide an explanation for the broad effects of hydrostatic pressure, which, as noted above, reduces actomyosin contractility and modulates cell-substrate adhesion through down-regulation of the MRTF/SRF pathway. Pretreatment at elevated pressure suppresses the viscosity-driven accumulation of nuclear YAP, which is responsible for establishing mechanical memory to viscosity (*35*), indicating that pressure alters not only how cells sense physical cues but also how they retain memory of them. Our findings may have important implications for cancer metastasis, as solid tumors typically exhibit elevated extravascular hydrostatic pressure and are subjected to compressive stresses (*3*, *12*, *14*), which we show can impose lasting effects on cellular mechanoperception. By losing mechanosensitivity, cancer cells could escape the primary tumor by migrating down stiffness and pressure gradients that exist between the tumor microenvironment and the surrounding tissue, evade fluid force–induced cell death during circulation, and form secondary tumors in mechanically diverse microenvironments. In addition, hydrostatic pressure may modulate cellular responses to chemical cues, as evidenced by its ability to increase resistance to the chemotherapy drug doxorubicin (*90*). Further studies are required to delineate both the direct and indirect effects of hydrostatic pressure on cellular function in health and disease, particularly as hydrostatic pressure remains a relatively understudied stimulus.

## MATERIALS AND METHODS

### Cell culture and reagents

Human MDA-MB-231 breast cancer cells (female donor; HTB-26) and HT-1080 fibrosarcoma cells (male donor; CCL-121) were acquired from American Type Culture Collection. MDA-MB-231-LifeAct-GFP cells, HT-1080-Paxillin-GFP, HT-1080-eGFP, and HT-1080-eGFP-RHOA-Q63L were gifted by K. Konstantopoulos. All cell lines were cultured in Dulbecco’s modified Eagle’s medium (DMEM; Gibco) supplemented with 10% (v/v) FBS Bio-Techne and 1% (v/v) penicillin/streptomycin (10,000 U/ml; Gibco) and maintained at 37°C in a humidified incubator with 5% CO_2_. Pharmacological treatments were carried out using the following drugs: CCG-1423 (S7719; 10 μM; Selleckchem), KPT-330 (S7252; 10 μM; Selleckchem), blebbistatin (2407-1; 25 μM; BioVision), Y-27632 (Y0503; 10 μM; Sigma-Aldrich), CK-666 (ALX-270-506-M002; 100 μM; Enzo Life Sciences), calyculin A (0215837810; 0.1 nM; MP Biomedicals), doxycycline (446060250; 0.05 μg/ml; Thermo Fisher Scientific) and LPA (89160-532; 50 μM; Enzo Life Sciences). MDA-MB-231-shSRF, MDA-MB-231-scramble, MDA-MB-231-SRF-VP16, MDA-MB-231-CNT, HT-1080-RhoA2G, and MDA-MB-231-RhoA2G cells were cultured as described above and maintained under continuous puromycin (J593-25MG; 1 μg/ml; VWR) selection.

### Photolithography and device fabrication

Device 1—2D microfluidic devices for pretreating cells under varying hydrostatic pressures: Microfluidic devices were fabricated containing a single channel with dimensions of *W* × *H* × *L* = 1 cm × 60 μm × 4 cm (Fig. 1A). Briefly, SU-8 (3025, Kayaku), a negative photoresist, was spin coated onto a silicon wafer to form the desired layer. Following ultraviolet (UV) exposure through a photomask with a 1-cm-wide channel pattern, the wafer was developed using SU-8 developer to remove the uncross-linked photoresist. PDMS and its curing agent were mixed thoroughly at a 10:1 ratio and degassed to eliminate air bubbles. The PDMS prepolymer was then poured over the microfabricated wafer and cured at 85°C for ~1 hour. After curing, the PDMS layer was plasma cleaned and bonded to a glass coverslip. Devices were subsequently coated with rat tail collagen type I (354236, Corning; 20 mg/ml) for 1 hour at 37°C to facilitate cell adhesion.

Device 2—microfluidic devices with an array of microchannels for measuring fluid force–induced channel entry: Microfluidic devices were fabricated containing an array of microchannels (Fig. 2A) with dimensions of *W* × *H* × *L* = 10 μm by 3 μm by 200 μm. Fabrication was carried out as described in our earlier work (*10*).

Device 3—trifurcating microfluidic devices for assessing cell decision-making: Microfluidic devices containing trifurcating Ψ-like microchannels (Fig. 2A) with dimensions as indicated in Table 1 were fabricated using the exact process as “Microfluidic devices with an array of microchannels for measuring fluid force–induced channel entry”. All dimensions were confirmed using a profilometer.

**Table 1.** Dimensions of the trifurcating microfluidic device.

Channel direction at the junction	Width (μm)	Height (μm)	Length (μm)	Relative hydraulic resistance
Left	10	3	300	1×
Straight	10	3	180	0.6×
Right	10	3	700	2.3×

### Cell exposure to varying hydrostatic pressure

System 1—prolonged cell exposure to varying hydrostatic pressures: Cells were resuspended in culture medium at a concentration of 70,000 cells per 40 μl. Cells were introduced into the collagen type I–coated 2D microfluidic device by adding a 40-μl aliquot of the suspension to one of its reservoirs. After flow equilibration, the device was then incubated at 37°C in a 5% CO_2_ humidified incubator for 15 min to permit cell attachment.

Hydrostatic pressure was subsequently applied by inserting long pipette tips into the reservoirs and adjusting the medium height, based on the gauge pressure equation *P*_gauge_ = ρ*gh*, where *P*_gauge_ refers to the pressure relative to atmospheric pressure, ρ is the density of water (1000 kg/m^3^), *g* is the gravitational acceleration (9.81 m/s^2^), and *h* is the height of the fluid column (Table 2). Hydrostatic pressure was maintained for 4 days unless otherwise stated, with medium changes every 18 to 24 hours to ensure a consistent supply of nutrients. Following the 4-day pretreatment, cells were exposed to varying physical cues as described in subsequent sections.

**Table 2.** Gauge pressures and the corresponding medium heights.

Gauge pressure (kPa)	~0.08	~0.12	~0.16	~0.3	~1	~3
Height (cm)	0.8	1.2	1.6	3.1	10.2	30.6

System 2—acute cell exposure to varying hydrostatic pressures: Cells were introduced into the 2D microfluidic devices as described in system 1. Following seeding, cells were allowed to adhere for 1 hour. Subsequently, medium-filled tubing was inserted into both the inlet and outlet reservoirs. The entire setup was then placed in an incubator, where a consistent hydrostatic pressure of 0.08 kPa was maintained across all conditions. Following this, the devices were transferred to a Nikon ECLIPSE Ti2 inverted microscope or Nikon Ti2 AX R confocal microscope equipped with a Tokai Stage-Top Incubator, which provides controlled CO_2_, temperature, and humidity. The setup was left undisturbed for 1 to 4 hours to allow the cells to acclimate to the new environment. During this period, cells were maintained under a hydrostatic pressure of 0.08 kPa. The tubing height of the devices was then adjusted to increase the hydrostatic pressure to 1 kPa (fig. S5B) or left unchanged to maintain a pressure of 0.08 kPa. This system was used to perform FRET experiments (Fig. 4, A and B, and fig. S5C) and MRTF-A localization studies (Fig. 4, C and D, and fig. S5, E to H).

### In vitro compression device

To apply a defined and controlled compressive stress to cells, a previously described transmembrane pressure device was used (*39*, *40*, *91*). Briefly, the setup involved a 24-mm transwell insert with 0.4-μm pores (Greiner Bio-One), into which MDA-MB-231 cells were seeded at a density of 2.5 × 10^5^ cells, 24 hours before compression (Fig. 2I). A 2% low–melting-point agarose gel was placed over the cell monolayer, and a piston of the desired weight was positioned on top of the gel to exert a compressive stress of 0.3 kPa for 2 days. Control cells (~0 Pa) were covered with agarose gel but not subjected to compression.

### Plasmids, lentivirus preparation, transduction, and plasmid transfection

The CArG-RE lentiviral dual promoter (LVDP) vector (Addgene plasmid #89762) and the lentiviral constructs encoding the puromycin resistance gene (control vector) or both SRF-VP16 and the puromycin resistance gene (SRF-VP16) were gifts from S. Andreadis (*59*). Experiments with cells expressing SRF-VP16 were performed after the pretreatment period and within 48 hours after transduction. The MRTF-A–GFP plasmid was a gift from M. K. Vartiainen (*58*). The nontargeting scramble control short hairpin RNA (shRNA) (GCACTACCAGAGCTAACTCAGATAGTACT) was kindly provided by K. Konstantopoulos (*92*). An shRNA plasmid targeting SRF (NM_003131, sequence: GAGACCGGCAAGGCACTGATT) was purchased from Sigma-Aldrich. Plasmids were amplified using One Shot Stbl3 (C737303, Invitrogen) Chemically Competent *Escherichia coli* and purified using a plasmid purification kit (12162, QIAGEN) as described by the manufacturer. Both lentiviral shRNA constructs were generated in a pLKO.1 puro backbone (plasmid# 8453, Addgene; a gift from B. Weinberg). Lentiviral particles were generated as described previously (*93*). For transduction, cells were incubated with lentiviral particles in the presence of polybrene (8 μg/ml; AB01643 AmericanBio) in DMEM supplemented with 10% (v/v) FBS and 1% (v/v) penicillin/streptomycin. HT-1080 cells were transfected with the MRTF-A–GFP plasmid using the Lipofectamine 3000 Transfection Kit (L3000008, Invitrogen) according to the manufacturer’s instructions.

### Quantification of MRTF-A–GFP

HT-1080 cells, transfected with MRTF-A–GFP, were seeded into 2D microfluidic devices as described in system 2. Devices were placed on a Nikon Ti2 AX R confocal microscope equipped with a 40× water immersion objective and a Tokai Stage-Top Incubator. Time-lapse images were acquired every 10 min for a total duration of 20 min. The nuclear-to-cytoplasmic ratio was then quantified from the captured images using the following equation

$$\text{Nuclear}/\text{cytoplasmic ratio}=\frac{I_{\text{nucleus}}-I_{\text{background}}}{I_{\text{cytoplasm}}-I_{\text{background}}}$$ (1)

where *I*_nucleus_ is the mean fluorescence intensity of the nucleus, *I*_cytoplasm_ is the mean fluorescence intensity of the cytoplasm, and *I*_background_ is the mean fluorescence intensity of the background. Image brightness was adjusted uniformly in all images for visualization purposes.

### FRET experiment

Cells stably expressing the RhoA2G biosensor were seeded in 2D microfluidic devices as described above in system 2. Fluorescence images were acquired using a Nikon ECLIPSE Ti2 inverted microscope equipped with a 40× air objective, cyan fluorescent protein (CFP; excitation: 440 nm, emission: 480/20 nm), YFP (excitation: 508 nm, emission: 540/20 nm), and FRET (excitation: 440 nm, emission: 540/20 nm) channels. In the control devices, imaging was first performed under a baseline pressure of 0.08 kPa, designated as time zero (*t* = 0 min), and repeated after 10 min. In the experimental device, cells were first imaged under a baseline pressure of 0.08 kPa at time *t* = 0 min. Subsequently, the pressure was increased to 1 kPa by adjusting the height of the tubes connected to the microfluidic device reservoirs (fig. S5B). The same cells were then imaged 10 min after the pressure was increased to assess their response to elevated hydrostatic pressure. The YFP channel image was used to delineate the cell boundary. To quantify RhoA activity, the mean fluorescence intensity ratio of FRET to CFP was calculated after subtracting background signals from their respective channels using ImageJ. For the experiment with LPA, cells were serum starved for 12 hours before treatment with LPA-containing medium in the absence of FBS. A vehicle control (VC) was included in parallel to account for any nonspecific effects of the treatment. Imaging was performed 10 min following the medium change.

### Fluid force–induced cell entry into confined microchannels and cell decision-making in trifurcating Ψ-like microchannels

After pretreatment, cells were trypsinized and harvested from the 2D microfluidic device. The collected cells were resuspended in culture medium at a concentration of 40,000 cells per 20 μl. A 20-μl aliquot of this suspension was then introduced into microchannel devices containing either an array of confining microchannels (device 2) or an array of trifurcating Ψ-like microchannels (device 3), as described previously (*10*, *38*). The devices were incubated at 37°C with 5% CO_2_ for ~30 min to allow the cells to adhere to the collagen I–coated surfaces.

To study fluid force–induced entry into confined microchannels (device 2), a pressure differential of −0.16 kPa was applied across the microchannels as described previously (*10*). Images were taken using a 10× air objective on a Nikon ECLIPSE Ti2 inverted microscope equipped with Tokai Stage-Top Incubator enabling CO_2_, temperature, and humidity control at *t* = 0 and at *t* = 6 hours. To quantify the percentage of cell entry, we defined a distance of 50 μm from the base of the microchannel, based on observations that cells more than 50 μm away rarely enter microchannels. The percentage of cell entry was calculated by counting the total number of cells that entered the microchannels and dividing it by the total number of cells within a 50-μm distance.

To monitor cell decision-making in the trifurcating device (device 3), time-lapse images were acquired every 10 min over an 18-hour period. Individual cells were manually tracked, and a decision was defined as the event in which the nucleus and at least 90% of the cell body entered a specific channel at the trifurcation.

### Memory experiment

Cells were preconditioned under low pressure (0.08 kPa) and high pressure (1 kPa) for 4 days. After this pretreatment, cells from each condition were collected and evenly divided into two groups. In the 0.08-kPa group, half of the cells were maintained at 0.08 kPa for an additional 4 days, while the other half was switched to 1 kPa for the same period. Similarly, in the 1-kPa group, one subgroup remained at 1 kPa, and the other was transferred to 0.08 kPa for 4 days. After this secondary conditioning phase, all groups were assessed for their responses to varying physical cues to evaluate the presence of mechanical memory.

### Preparation of high-viscosity medium

Iscove’s Modified Dulbecco’s Medium (822, Gibco) was supplemented with 1% (v/v) penicillin/streptomycin and 10% (v/v) FBS. A 3% methylcellulose stock solution (HSC001, R&D Systems) was then incorporated using a syringe to achieve a final methylcellulose concentration of 0.6%. The average viscosity of the resulting solution, measured at 37°C using an Anton Paar MCR301 rotational rheometer, was 11.2 cP (*10*). The medium was subsequently filtered through a 0.2-μm pore-size filter and stored at 4°C for future use.

### Hypoxia pretreatment

To evaluate hypoxia-induced changes in mechanosensitivity, cells were seeded into 2D microfluidic devices (system 1) and cultured at low pressure (0.08 kPa) under either normoxic (21% O_2_) or hypoxic (1% O_2_) conditions for 4 days. Medium was replaced every 18 hours, and before each change, fresh medium was preequilibrated for 4 hours in either the normoxic or hypoxic incubator.

Following the 4-day treatment, an oxygen indicator (Image-iT Green Hypoxia Reagent, #I14833, Invitrogen) was added to the devices at a final concentration of 5 μM. Cells were incubated with the indicator under either normoxic or hypoxic conditions for 3 hours and then washed three times with phosphate-buffered saline (PBS). Imaging was performed using an inverted Nikon ECLIPSE Ti2 microscope equipped with a 20× air objective. Hypoxia-associated fluorescence intensity was quantified as follows

$$\begin{array}{c} \text{Integrated intensity}\;\text{per}\;\text{cell}=\text{total intensity}\;\text{per}\;\text{cell}\\ -\left(\text{cell area}\times \text{mean background intensity}\right) \end{array}$$ (2)

### Live-dead staining to quantify cell viability

To assess cell viability, cells were introduced into 2D microfluidic devices and exposed to low (0.08 kPa) or high (1 kPa) pressure, as described above in system 1. After 4 days of treatment, cell viability was evaluated using a live-dead staining kit (K502-100-2, BioVision), following the manufacturer’s protocol. The percentage of cells that remain viable was calculated using the following equations

$$\text{Cell death}=\frac{\text{number of dead cells}\;\left(\text{red}\;\text{cells}\right)}{\text{total number of cells}}$$ (3)

%Cell viability=(1−cell death)×100 (4)

### Preparation of polyacrylamide gels

Coverslips were heated to 1080°F, followed by treatment with 0.1 N NaOH (BDH7222-1, VWR) and air drying for 10 min under a biosafety hood. Each coverslip was then coated with 3-aminopropyltrimethoxysilane (281778-100ML, Sigma-Aldrich), incubated under the biosafety hood for 30 min, and rinsed with deionized water (dH_2_O) at room temperature. Following this, the coverslips were treated with 0.5% (v/v) glutaraldehyde (G7776-10ML, Sigma-Aldrich) for 30 min, washed three times with dH_2_O, and air dried.

A prepolymer solution was prepared by mixing (A) acrylamide (40%; M121-500ML, VWR) and (B) *N*,*N*′-methylene-bis-acrylamide (2%; 1610142, Bio-Rad) together with ammonium persulfate (10%; 1610700, Bio-Rad), *N*,*N*,*N*′,*N*′-tetramethylethylenediamine (161-0800, Bio-Rad), and Hepes buffer (J848-100ML, VWR). The concentrations of acrylamide and bis-acrylamide were adjusted to achieve varying gel stiffness (see Table 3). The prepolymer mixture was applied to the activated coverslips, overlaid with untreated coverslips to ensure uniform thickness, and allowed to polymerize for ~30 min.

**Table 3.** Concentrations of acrylamide and bis-acrylamide, and the corresponding stiffness.

Sample	A (%)	B (%)	Average Young’s modulus (kPa)
PA1	3	0.2	0.37
PA2	8	0.6	5.36
PA3	11	0.5	12.87
PA4	15	1.2	20.18

After polymerization, the gels were incubated at 37°C for 3 days in PBS to allow for swelling. To coat the polyacrylamide surfaces with ECM proteins, the hydrogels were treated with sulfosuccinimidyl-6-(4′-azido-2′-nitrophenylamino)hexanoate (2 mg/ml; 35395, Thermo Fisher Scientific), exposed to UV light (254 nm) for 15 min, and incubated with rat tail collagen I (20 μg/ml) at 37°C for 1 hour.

### Hydrogel stiffness measurement via nanoindentation

Nanoindentation (FT-MTA03, FemtoTools) was used to determine the Young’s modulus of the polyacrylamide gels following a previously established protocol (*94*). Briefly, a spherical ruby-tipped probe with a diameter of 125 μm was used to indent each hydrogel sample (*n* = 3) a total of 25 times using a 5 × 5 array. The recorded force and displacement data were plotted and subsequently fit to the Oliver-Pharr model (*94*, *95*).

### Immunocytochemistry

Cells preconditioned for 4 days at different hydrostatic pressures (as described in system 1) or for 2 days under varying compressive stresses (as detailed in the section In vitro compression device) were seeded onto collagen type I–coated polyacrylamide hydrogels of varying stiffness or onto glass slides where they were exposed to fluids with different viscosities. After 4 hours, cells were fixed with 4% paraformaldehyde for 10 min, followed by three washes with PBS. Permeabilization was carried out by incubating cells with 0.1% (v/v) Triton X-100 for 15 min and then washing with PBS to remove residual detergent. To minimize nonspecific binding, cells were blocked for 20 min using a blocking buffer containing 2% (v/v) goat serum, 0.1% (v/v) Triton X-100, and 5% (v/v) bovine serum albumin (BSA) in PBS.

Primary antibodies [anti-YAP (sc-101199, Santa Cruz, RRID:AB_1131430; 1:50), anti–pMLC2 (Ser^19^; 3671, Cell Signaling Technology, RRID:AB_330248; 1:200), and anti-paxillin (50195T, Cell Signaling Technology, RRID: AB_3698765; 1:200)] were diluted in blocking buffer and applied to cells overnight at 4°C. The following day, cells were washed with PBS to remove unbound antibodies and incubated with secondary antibodies [Alexa Fluor 488 goat anti-mouse (A11029, Invitrogen, RRID:AB_253408; 1:100) or Alexa Fluor 488 goat anti-rabbit (A11034, Invitrogen, RRID:AB_2576217; 1:100) and Hoechst (40046, Biotium; 1:200) for 2 hours. Fluorescence imaging was conducted using a Nikon ECLIPSE Ti2 inverted microscope equipped with a 20× air objective (Figs. 2, D and E, 3A, and 4G), a Nikon Ti2 AX R confocal microscope equipped with 40× water (Figs. 1, F and G, 3, D and E, and 4, F and H; and fig. S1, H and I) and 60× oil (Fig. 2C and fig. S2C) immersion objectives or a STELLARIS 5 confocal microscope equipped with 60× oil immersion objective (Fig. 2J and fig. S3, A and B). Image brightness was adjusted uniformly in all images for visualization purposes. The YAP analysis was conducted in a manner similar to the quantification of MRTF-A nuclear-to-cytoplasmic ratio. pMLC levels were quantified similarly to hypoxia fluorescence intensity. pMLC in selected experiments was analyzed in a blinded manner.

### Western blot

shSRF and scramble control MDA-MB-231 cells were lysed using radioimmunoprecipitation assay buffer (89901, Thermo Fisher Scientific) supplemented with phosphatase inhibitor (78428, Thermo Fisher Scientific) and protease inhibitor (87785, Thermo Fisher Scientific) to prevent protein degradation. Following lysis, total protein concentrations were quantified using the BSA Protein Assay Kit (5000207, Bio-Rad) and Quick Start Bradford 1× Dye Reagent (5000205, Bio-Rad). Lysates were either stored at −80°C or used immediately.

A total of 30 μg of total protein from each sample (scramble and shSRF) was mixed with 2× Laemmli Sample Buffer (1610737, Bio-Rad), reduced with 2-Mercaptoethanol (M3148-25ml, Sigma-Aldrich), and heated at 95°C for 10 min to denature proteins. Proteins were then separated by SDS–polyacrylamide gel electrophoresis using 4–15% Mini-PROTEAN TGX Precast Protein Gels with a 10-well comb (4561084, Bio-Rad). After electrophoresis, proteins were transferred onto nitrocellulose membranes (1704159, Bio-Rad) using the Trans-Blot Turbo Transfer System (Bio-Rad). Membranes were blocked for 1 hour at room temperature in 5% (w/v) BSA prepared in Tris-Buffered Saline with Tween-20 (TBST) and incubated overnight at 4°C with SRF Rabbit Monoclonal Antibody (5147, Cell Signaling Technology, RRID:AB_10694554) diluted 1:1000 in 5% (w/v) BSA. The following day, membranes were incubated for 60 min at room temperature with horseradish peroxidase (HRP)–linked anti-rabbit immunoglobulin G secondary antibody (7076S, Cell Signaling Technology, RRID:AB_330924), diluted 1:1000 in blotting-grade nonfat dry milk (1706404, Bio-Rad) prepared in TBST. Chemiluminescent signals were detected using Amersham ECL detection reagent (89233-310, VWR) and visualized with an Azure 300 using a 60-s exposure.

### Quantification of FA

Acquired images of paxillin were imported to ImageJ/Fiji and converted to 8-bit grayscale. The processing of FAs on a 2D substrate was conducted using a previously described method (*96*). To remove uneven fluorescence, the background of the acquired images was subtracted using the Sliding Paraboloid method with a rolling-ball radius of 50. This step was followed by enhancing local contrast using contrast limited adaptive histogram equalization (block size: 19, histogram bins: 256, max slope: 6, fast, no mask). The Exp plugin was then applied to suppress low background levels. Last, the processed images were converted to binary format using Auto Threshold (default settings) and analyzed to determine the number and size of FAs using the Analyze Particles plugin.

### Quantification of CArG-RE

To quantify CArG-RE activity, cells stably expressing the reporter construct were treated with VC or CCG-1423 or seeded in 2D devices and exposed to different levels of hydrostatic pressure. On the third day, cells were trypsinized and replated to minimize the impact of confluency on transcriptional activity. After 4 days of pretreatment, cells were imaged using fluorescein isothiocyanate and tetramethyl rhodamine isothiocyanate filters on an inverted Nikon ECLIPSE Ti2 microscope with a 10× air objective. Analysis of the images was done using the ImageJ software. The ratio of green to red fluorescence intensity (GFI and RFI) was calculated using the formula below

$$\text{CArG-RE}\;\text{activity}=\frac{I_{\text{GFI},\text{image}}-I_{\text{GFI},\text{background}}}{I_{\text{RFI},\text{image}}-I_{\text{RFI},\text{background}}}$$ (5)

where I*_GFI,image_* is the mean GFI of the image, *I*_GFI,background_ is the mean GFI of the background, *I*_RFI,image_ is the mean RFI of the cell, and *I*_RFI,background_ is the mean RFI of the background. Data were normalized to VC or 0.08 kPa. Two repeats of CArG-RE activity in MDA-MB-231 cells were analyzed in a blinded manner.

### Cell protrusion growth

After 4 days of pretreatment under 1-kPa hydrostatic pressure using system 1, MDA-MB-231–LifeAct-GFP cells were seeded on hydrogels of 13 or 20 kPa. After 4 hours, images were taken every 1 min for 30 min using a Nikon Ti2 AX R confocal microscope equipped with ×40 water immersion objective. Using the line tool in ImageJ, a straight-line region of interest (ROI; 30 μm) was drawn perpendicular to where active lamellipodia protrusion was observed at the cell leading edge, and the kymograph function was applied. This created a 2D kymograph plot.

A Python script was developed to track the morphology of the cells over time in the time-lapse microscopy images. This code began by extracting .nd2 frames into the PNG format for downstream processing and normalized each frame with a MinMaxScaling routine based on grayscale pixel intensity to preserve contrast between pixels across frames. Once frames of the .nd2 file are extracted, we manually define a single ROI within the first frame and thereby a specific cell to analyze throughout the time series. This ROI selection is manually completed with a graphical user interface and works best when analyzing relatively stationary cells. Each time frame of the series is then cropped to the selected ROI to reduce pixel image data, and these cropped images are used for future analyses. The Python Open Source Computer Vision library is used to open each cropped ROI, apply Gaussian filtering to suppress noise, and apply an Otsu thresholding to binarize the image (*97*). From this binary map, the contour with the largest area is assumed to be the cell of interest, and the shape of the contour is saved per frame to a data file. This assumption relies on the ROI being chosen such that it is primarily occupied by the specific cell of interest. These contours are taken directly from the binarized image map and contain jaggedness due to the pixel discretization. As a result, a smoothing algorithm is applied to these contours using a SciPy b-spline function (*98*). Once a smoothed contour has been produced for each time frame, these contours are plotted over the initial time frame image to form a color-coded pixel map of cell boundaries within the selected ROI (*99*).

### Quantification of 2D cell migration and morphological features

2D cell migration was monitored using an inverted Nikon ECLIPSE Ti2 microscope equipped with a 10× Ph1 objective. Time-lapse imaging was performed over 8 hours, with images captured every 10 to 22 mins. The resulting image stacks were exported to ImageJ, and the MTrackJ plugin was used to manually track cell motility throughout the experiment.

We used a custom MATLAB (MathWorks) script to extract cell migration speed on 2D substrates from the persistent random walk (PRW) model, which has been widely applied to describe 2D cell migration under conditions where no symmetry-breaking gradients (e.g., chemotactic cues) are present (*100*, *101*). Specifically, the mean-squared displacement (MSD) of each cell was calculated as a function of the time lag τ based on experimentally acquired trajectories using the following equation

$$\text{MSD}\left(\tau \right)=\left\langle {\left[x\left(t+\tau \right)-x\left(t\right)\right]}^{2}+{\left[y\left(t+\tau \right)-y\left(t\right)\right]}^{2}\right\rangle$$ (6)

where *x*(*t*) and *y*(*t*) are the coordinates of the cell and x(t+τ) and y(t+τ) are the coordinates of the same cell after a time lag τ. We focused exclusively on single cells that neither divided nor interacted with neighboring cells. The MSD of a cell following a random walk is also given by

$$\text{MSD}\left(\tau \right)=nS^{2}P\left[\tau -P\left(1-e^{-\frac{\tau }{P}}\right)\right]+4\sigma^{2}$$ (7)

where n is a dimensionality constant (*n* = 2 for 2D migration), S is the speed, P is the persistence time, and $\sigma^{2}$ represents experimental noise (*102*). By fitting Eq. 7 to the experimental MSD series, we extracted migration speed of individual cells. Following the protocols established by Wu *et al.* (*102*), only the first 50% of each cell’s MSD series was used for model fitting to minimize statistical noise at high lag times.

The Polygon Selection tool in ImageJ was manually used to delineate the periphery of each cell, creating ROIs for morphological analysis. Each ROI was subsequently measured to extract morphological parameters, such as cell area and aspect ratio. All analyses were conducted blindly: Experimental conditions were labeled alphabetically and assigned to an independent analyst who had no involvement in the experiments and no prior knowledge of the condition identities.

### Cell trajectory plots

To visually depict cell trajectories, the *x* and *y* coordinates of manually tracked cells over the course of the experiment were obtained using the MTrackJ plugin, as described above. For each cell, the *x* and *y* coordinates at time 0 were subtracted from all subsequent positions to align the starting point of each trajectory at the origin (0,0). The normalized coordinates were then used to generate trajectory plots using a custom MATLAB script.

### An actomyosin-based model for cell migration

This model considers steady-state cell migration in a 1D space along the x direction. We let $x^{f}$ be the front of the cell, whereas $x^{b}$ is the back of the cell so that $L=x^{f}-x^{b}$ is the length of the cell. Under steady state, the cell length is a constant and the cell moves at a constant velocity $v_{\text{cell}}$. Throughout the model, we will use the scripts “f” and “b” to denote quantities associated with the front and back of the cell, respectively. This model focuses on actin and its interaction with the substrate. Other components such as cytosol, water flux, electrolytes, and osmosis are irrelevant to the scientific question we study in this work and thus are not included. Interested readers can refer to our earlier works for models that include additional modulus of multiphase cell migration (*103*–*105*).

The F-actin network is mechanically connected to FAs to provide the driving force for cell migration. The presence of actin filaments creates a passive swelling stress, $\sigma_{n}$, which is modeled by a linear constitutive relation, $\sigma_{n}=k_{\sigma_{n}}\theta_{n}$, where $k_{\sigma_{n}}$ is the coefficient of actin swelling and $\theta_{n}\left(x\right)$ is the concentration of F-actin.

The conservation of momentum for the F-actin is

$$\frac{\partial \sigma }{\partial x}+\eta_{\text{st}}\theta_{n}v_{n}=0$$ (8)

where $v_{n}$ is the velocity of F-actin and $\eta_{\text{st}}$ is the strength of FA. The force from FA, $\eta_{\text{st}}\theta_{n}v_{n}$, is modeled as an effective body force on the actin network (*106*); this term makes a mechanical connection between the F-actin network and the FA. The strength of FA, $\eta_{\text{st}}$, indicates the coefficient of force transmission from the FA to the actin network per unit amount of actin network momentum, $\theta_{n}v_{n}$. It depends on the substrate stiffness, $k_{\text{st}}$, and is modeled as

$$\eta_{\text{st}}=\eta_{\text{st},0}+\eta_{\text{st},m}{\left(\frac{k_{\text{st}}}{k_{\text{st},0}}\right)}^{2}$$ (9)

where $\eta_{\text{st},0}$ is a baseline strength of FA, $k_{\text{st},0}$ is a normalization factor, and $\eta_{\text{st},m}$ is the coefficient of FA influenced by actomyosin network engagement. In this model, we treat $\eta_{\text{st},m}$ as a parameter to quantify the influence of myosin on the strength of FA. Disruption of myosin activity reduces this coefficient.

The conservation of mass for F-actin is

$$\frac{\partial \theta_{n}}{\partial t}+\frac{\partial }{\partial x}\left(\theta_{n}v_{n}\right)=J_{\text{actin}}\;-\;\gamma \theta_{n}$$ (10)

where γ is the rate of actin depolymerization, which accounts for the interchange between F-actin and G-actin (*107*). $J_{\text{actin}}$ is the rate of actin polymerization which depends on the availability of G-actin but also saturates at large limit of G-actin concentration. This dependence can be described by

$$J_{\text{actin}}=J_{a}^{0}\left(\frac{\theta_{c}}{\theta_{c,c}+\theta_{c}}\right)$$ (11)

where $J_{a}^{0}$ is the coefficient of actin polymerization, $\theta_{c}\left(x\right)$ is the concentration of G-actin, and $\theta_{c,c}$ is a constant. The boundary conditions for the actin network follows the conservation of mass at the boundaries

$${\left(\theta_{n}v_{n}\right)}_{x=x^{f}}=\theta_{n}^{f}v_{\text{cell}}$$ (12)

The diffusion-reaction equation for the G-actin phase is

$$\frac{\partial \theta_{c}}{\partial t}=D_{\theta_{c}}\frac{\partial^{2}\theta_{c}}{\partial x^{2}}-J_{\text{actin}}+\gamma \theta_{n}$$ (13)

where $D_{\theta_{c}}$ is the diffusion coefficient of G-actin in the cytoplasm. The boundary conditions for G-actin are

$${\left(-D_{\theta_{c}}\frac{\partial \theta_{c}}{\partial x}\right)}_{x=x^{f}}=\theta_{c}^{f}v_{\text{cell}},\;\;{\left(-D_{\theta_{c}}\frac{\partial \theta_{c}}{\partial x}\right)}_{x=x^{b}}=\theta_{c}^{b}v_{\text{cell}}$$ (14)

The total actin is conserved such that

$$\int_{x^{b}}^{x^{f}}\left(\theta_{n}+\theta_{c}\right)\mathrm{dx}=L\theta_{*}$$ (15)

where $\theta_{*}$ is the average concentration of total actin. The force balance of the entire cell is

$$\eta_{\text{st}}\int_{x^{b}}^{x^{f}}\theta_{n}v_{n}\mathrm{dx}+k_{\text{ad}}v_{\text{cell}}=0$$ (16)

where $k_{\text{ad}}$ is the coefficient of frictional force on the cell. Solving cell velocity gives

$$v_{\text{cell}}=-\frac{\eta_{\text{st}}}{k_{\text{ad}}}\int_{x^{b}}^{x^{f}}\theta_{n}v_{n}\mathrm{dx}$$ (17)

This equation indicates that the driving force of cell migration comes from the force transmitted by the FA ($\eta_{\text{st}}$) to the flowing actin network ($v_{n}$), which is generated through actin polymerization. As the model considers 1D cell motility without bidirectional movement, velocity is equivalent to speed. The model parameters can be found in Table 4.

**Table 4.** Model parameters.

Parameters	Description	Values	Sources
*L* (μm)	Cell length	50	Convention
$\eta_{\text{st},0}$ (Pa⋅s/μm^2^/mM)	Baseline focal adhesion strength	2 × 10^2^	(*104*, *106*)
$\eta_{\text{st},m}$ (Pa⋅s/μm^2^/mM)	Actomyosin-induced focal adhesion coefficient	1 × 10^4^	Fitted
$k_{\text{st},0}$ (kPa)	Reference stiffness of the substrate	10	Estimated
$k_{\sigma_{n}}$ (Pa/mM)	Passive F-actin stress coefficient	1 × 10^4^	(*108*)
$k_{\text{ad}}$ (Pa⋅s/μm)	Coefficient of adhesive force	2 × 10^3^	Fitted
$\theta_{c,c}$ (μM)	Constant in actin polymerization	0.2	(*108*)
$\theta_{*}$ (mM)	Average concentration of totally actin	0.3	(*109*, *110*)
$J_{a}^{0}$ (μM/s)	Coefficient of actin polymerization	0.8	Fitted
γ (1/s)	Rate of actin depolymerization	4 × 10^−3^	Estimated
$D_{\theta_{c}}$ (μm^2^/s)	Diffusion coefficient of G-actin	10	(*105*)

### Artificial intelligence–assisted technologies

ChatGPT (version GPT-5 and 5.2) and Grammarly for Microsoft Office (version 6.7.205) were used during manuscript preparation using the following prompt: “Refine this text by correcting grammatical issues and improving syntax and structure.”

### Statistical analysis

All experiments were conducted using at least three independent biological replicates, unless otherwise noted. The D’Agostino-Pearson omnibus test was used to assess whether the data followed a normal distribution. The following statistical tests were used to determine statistical significance (*P* < 0.05): paired and unpaired *t* tests, one-way analysis of variance (ANOVA) followed by Tukey’s multiple-comparison test, and two-way ANOVA followed by Tukey’s multiple-comparison test, if the datasets followed normal distribution. For datasets that did not follow a normal distribution, log transformation was done before comparison. In cases where datasets did not conform to either normal or log-normal distribution, nonparametric tests were applied. The Kruskal-Wallis test followed by Dunn’s post-hoc test was used for comparisons involving more than two groups, whereas two-tailed Mann-Whitney tests were used for comparisons between two groups. All analyses were performed using the GraphPad Prism 10.6 software.
