# Supplementary material for: Hydrostatic pressure reduces the mechanosensitivity of cell migration
Source: Sci Adv. 2026 Jun 26;12(26):eaed0981. doi: 10.1126/sciadv.aed0981 (PMC13308595; doi:10.1126/sciadv.aed0981)
Supplement: Supplementary file 1 — Figs. S1 to S6 Legends for movies S1 to S4 [file sciadv.aed0981_sm.pdf]

Science Advances
AAAS

Supplementary Materials for

Hydrostatic pressure reduces the mechanosensitivity of cell migration

Ayuba Akinpelu *et al.*

Corresponding author: Panagiotis Mistriotis, pmistriotis@auburn.edu

Sci. Adv. **12**, eaed0981 (2026)
DOI: 10.1126/sciadv.aed0981

The PDF file includes:

Figs. S1 to S6
Legends for movies S1 to S4

Other Supplementary Material for this manuscript includes the following:

Movies S1 to S4

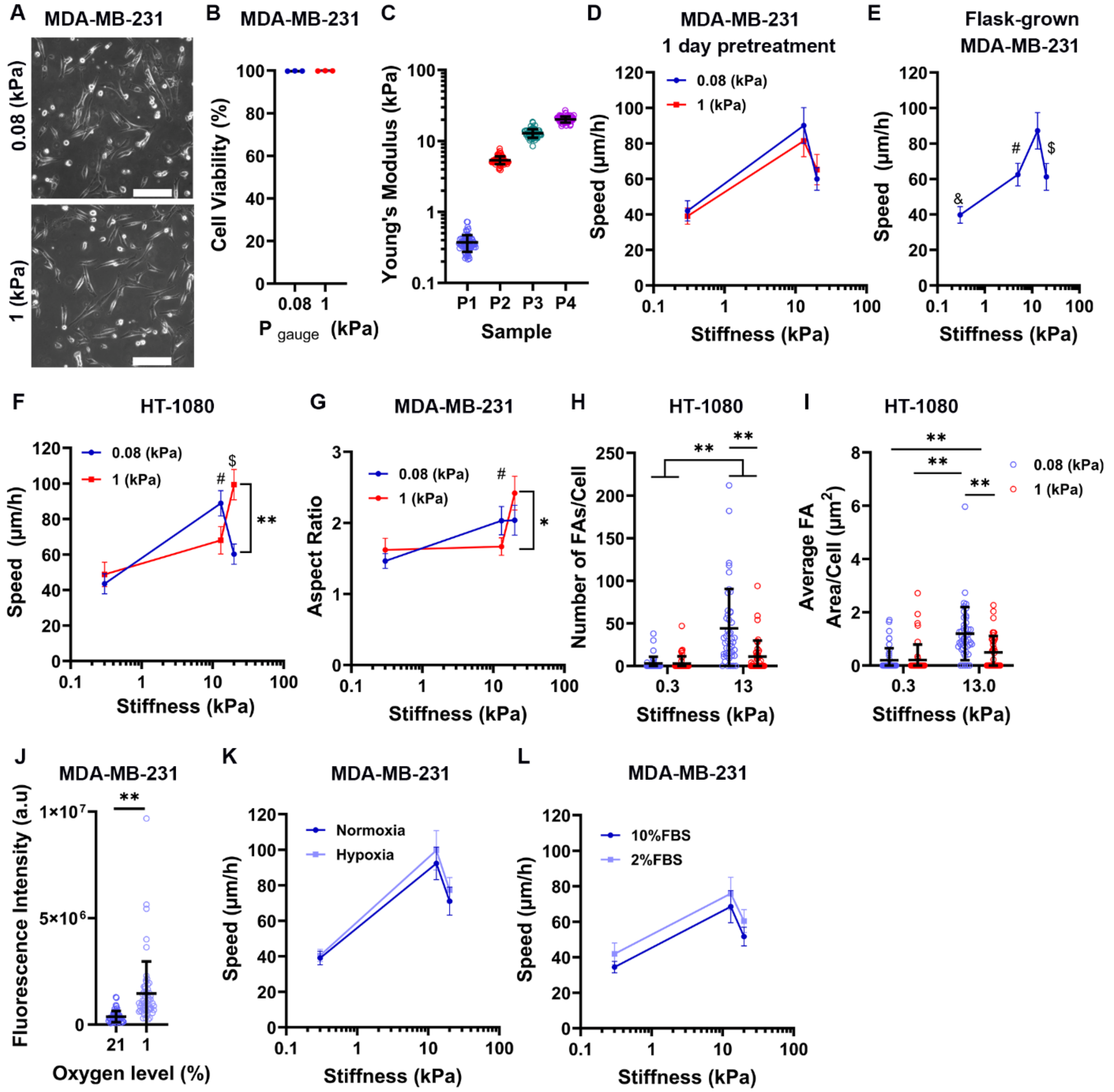

Fig. S1. Hydrostatic pressure-induced loss of stiffness sensing is independent of hypoxia or nutrient depletion. (A) Representative images of MDA-MB-231 cells under 0.08 or 1 kPa hydrostatic pressure for four days. Scale bar: 100 µm. **(B)** Percentage of MDA-MB-231 cells that remain viable after four days of culture under 0.08 or 1 kPa hydrostatic pressure (≥1000 cells analyzed/experiment, N=3). **(C)** Young's modulus of distinct PA gels prepared using different acrylamide to bis-acrylamide ratios and measured via the nanoindentation technique (25 distinct positions arranged in a 5 × 5 grid, N = 3 samples/condition). **(D-E)** Speed of MDA-MB-231 cells pretreated for one day at 0.08 or 1 kPa **(D)** or directly from flasks **(E)** on substrates of different stiffness (n=90/condition, N=3). $^{\&,\$,\#}p < 0.01$ (& 0.3 kPa vs. 5 kPa; # 5 kPa vs. 13 kPa, $ 13 kPa vs. 20 kPa). **(F)** Speed of pretreated (0.08 kPa or 1 kPa) HT-1080 cells on substrates of different stiffness (n=120/condition, N=4). $^{**,\$,\#}p < 0.01$ ($ 1 kPa cells on 13 kPa vs. 1 kPa cells on 20 kPa; # 0.08 kPa cells on 13 kPa vs. 1 kPa cells on 13 kPa). **(G)** Aspect ratio of pretreated (0.08 kPa or 1 kPa) MDA-MB-231 cells on substrates of different stiffness (n=105/condition, N=3). $^{*}p < 0.05$,

$^{\#}p < 0.01$ (# 0.08 kPa cells on 13 kPa vs. 1 kPa cells on 13 kPa). **(H-I)** FA number **(H)** and FA average area **(I)** in pretreated (0.08 kPa or 1 kPa), Paxillin-GFP labeled HT-1080 cells on substrates of different stiffness (n=45/condition, N=3). $^{**}p < 0.01$. **(J)** Fluorescence intensity of the oxygen indicator Image-iT in MDA-MB-231 cells cultured for four days under 0.08 kPa in hypoxic (1% v/v O_2) or normoxic (21% v/v O_2) environments (n=60/condition, N=3). $^{**}p < 0.01$. **(K)** Speed of pretreated (0.08 kPa with 1% or 21% v/v O_2) MDA-MB-231 cells on substrates of different stiffness (n=90/condition, N=3). **(L)** Speed of pretreated (0.08 kPa with 2% or 10% v/v FBS) MDA-MB-231 cells on substrates of different stiffness (n=90/condition, N=3). Pretreatments (**F-I and K,L**): four days. Statistics: unpaired t-tests **(B)**, Kruskal–Wallis test followed by Dunn's test **(E)**, two-way ANOVA followed by Tukey's multiple comparison test **(D, F-I, K, L)**, and Mann–Whitney test **(J)**. Values: mean ± SEM **(B)**, mean ± SD **(C, H-J)**, and mean ± 95% CI **(D-G, K, L)**.

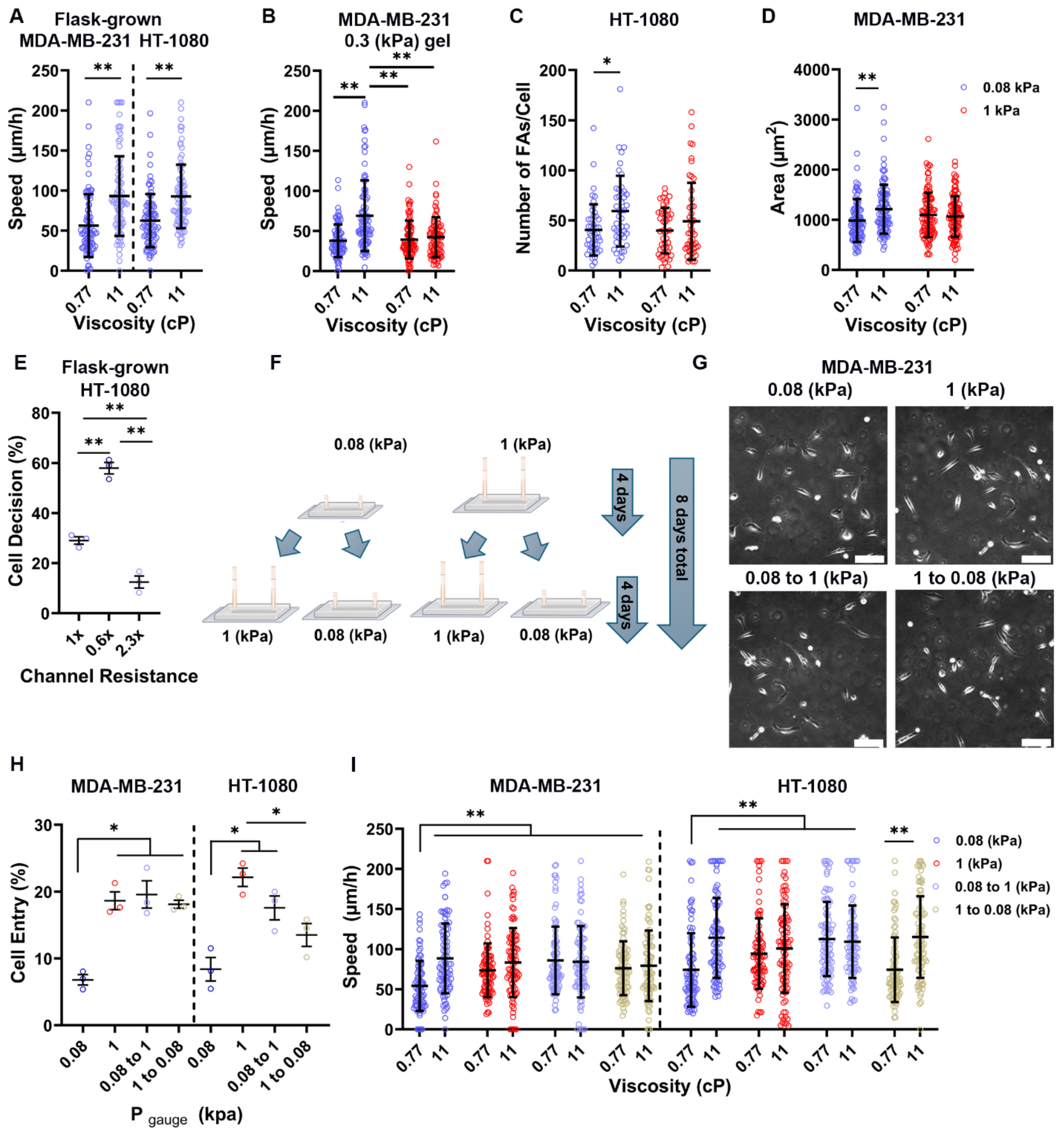

Fig. S2. Migrating cells retain a memory of past exposure to elevated hydrostatic pressure. **(A)** Speed of flask-grown MDA-MB-231 or HT-1080 cells in media of different viscosities (n=90/condition, N=3). $^{**}p < 0.01$. **(B)** Speed of pretreated (0.08 kPa or 1 kPa) MDA-MB-231 cells on soft (0.3 kPa) gels in media of different viscosities (n=90/condition, N=3). $^{**}p < 0.01$. **(C)** FA number in pretreated (0.08 kPa or 1 kPa), Paxillin-GFP-labeled HT-1080 cells on glass slides in media of different viscosities (n=45/condition, N=3). $^{*}p < 0.05$. **(D)** Projected area of pretreated (0.08 kPa or 1 kPa) MDA-MB-231 cells on glass slides in media of different viscosities (n=105/condition, N=3). $^{**}p < 0.01$. **(E)** Distribution pattern of flask-grown HT1080 cells in microchannels of different hydraulic resistances (>60 cells analyzed/experiment, N=3). $^{**}p < 0.01$. **(F)** Schematic of the experimental design to assess memory to hydrostatic pressure. **(G)**

Representative images of MDA-MB-231 cells under 0.08 kPa or 1 kPa for eight days or under 0.08 kPa or 1 kPa for four days followed by exposure to 1 kPa or 0.08 kPa for an additional four days. Scale bar: 100 μm. **(H)** Percentage of MDA-MB-231 or HT-1080 cells that enter confined microchannels under flow (ΔP=-0.16 kPa) conditions after continuous eight-day pretreatment at 0.08 kPa or 1 kPa or after four-day pretreatment at 0.08 kPa or 1 kPa, followed by four days under the alternate pressure (>100 cells analyzed/experiment, N≥3). *$p < 0.05$. **(I)** Speed of MDA-MB-231 or HT-1080 cells in media of different viscosities after continuous eight-day pretreatment at 0.08 kPa or 1 kPa or after four-day pretreatment at 0.08 kPa or 1 kPa, followed by four days under the alternate pressure (n=90/condition, N=3). **$p < 0.01$. Pretreatments **(B–D)**: four days. Statistics: Mann–Whitney tests **(A)**, two-way ANOVA followed by Tukey's multiple comparison test **(B-D, I)**, and one-way ANOVA followed by Tukey's multiple comparison test **(E, H)**. Values: mean ± SD **(A-D, I)**, mean ± SEM **(E, H)**.

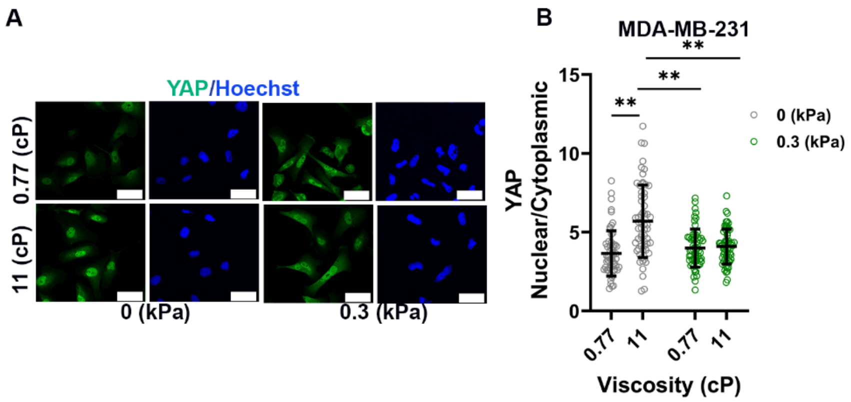

Fig. S3. Prolonged cell compression diminishes mechanosensing. (A-B) Representative images **(A)** and quantification of nuclear-to-cytoplasmic ratio of YAP **(B)** in MDA-MB-231 cells pre-exposed to ~0 kPa or 0.03 kPa using the transmembrane pressure device, measured on glass slides in media of different viscosities (n=60/condition, N=3). Scale bar: 100 µm. $^{**}p < 0.01$**.** Pretreatments: 2 days. Statistics: two-way ANOVA followed by Tukey's multiple comparison test. Values: mean ± SD.

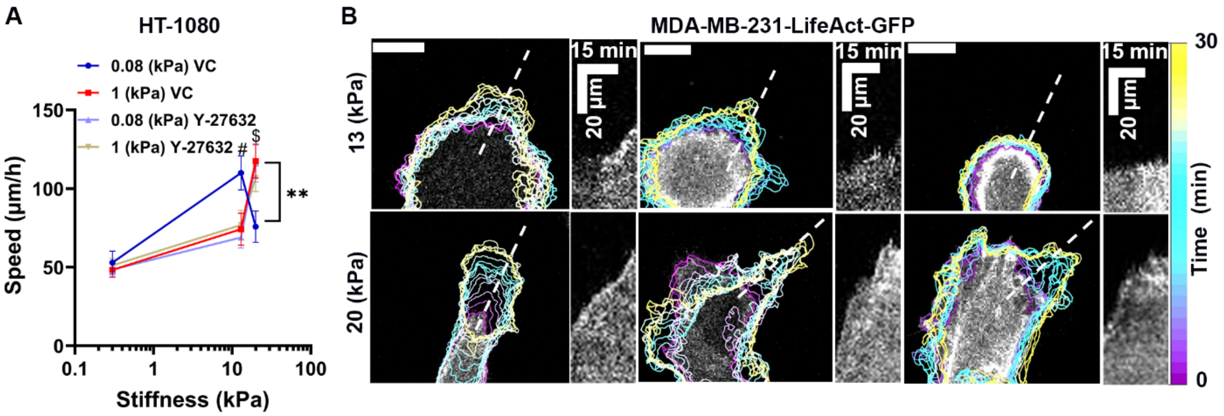

Fig. S4. Hydrostatic pressure reprograms the stiffness-sensing machinery. (A) Speed of preconditioned (0.08 kPa or 1 kPa) HT-1080 cells, then treated with VC or Y-27632 (10 µM) on substrates of different stiffness (n=90/condition, N=3). $^{**,\$,\#}p < 0.01$. ($ 1 kPa VC, 0.08 kPa Y-27632 and 1 kPa Y-27632 cells on 13 kPa vs. same conditions on 20 kPa; # 0.08 kPa VC on 13 kPa vs. 1 kPa VC, 0.08 kPa Y-27632 and 1 kPa Y-27632 on 13 kPa). **(B)** Additional contours (scale bar: 35 µm) and kymographs of pretreated (1 kPa), LifeAct-GFP-labeled MDA-MB-231 cells on substrates of different stiffness. Pretreatments: four days. Statistics: two-way ANOVA followed by Tukey's multiple comparison test. Values represent mean ± 95% CI.

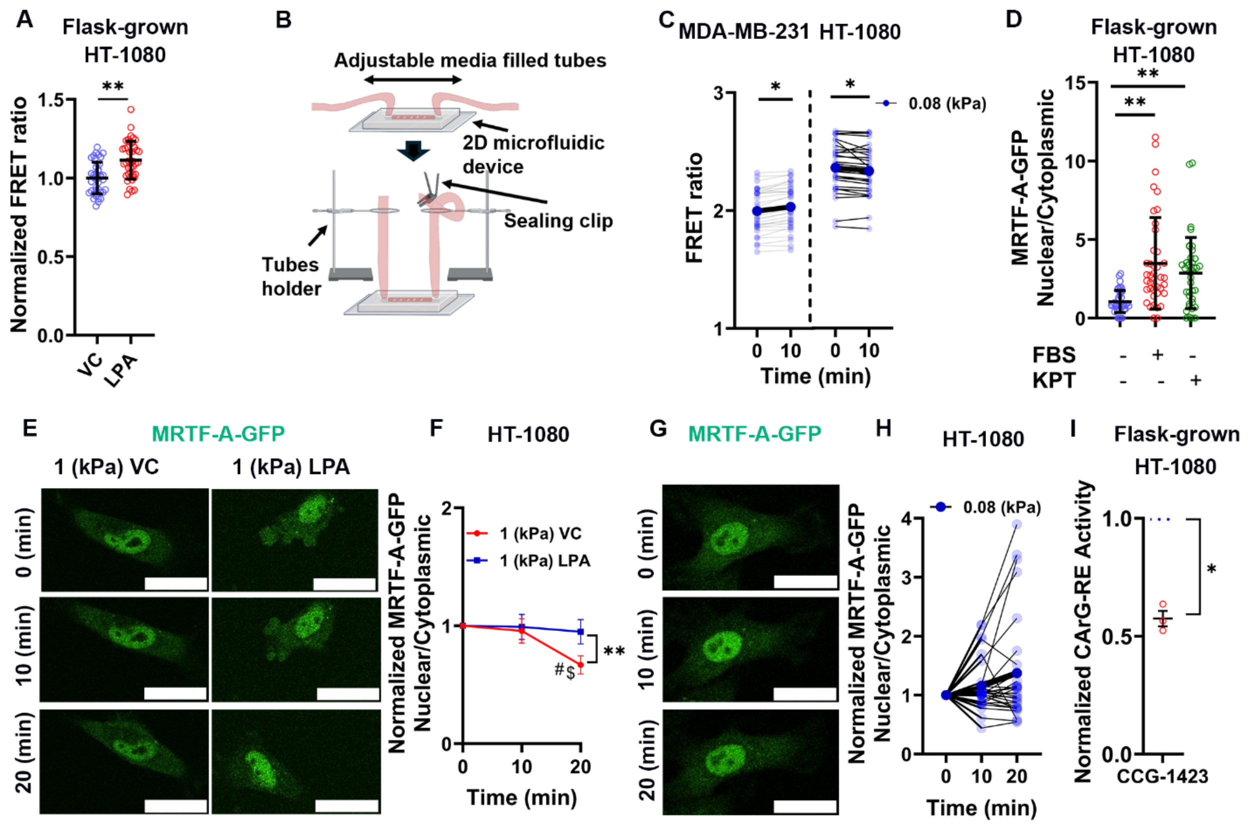

Fig. S5. Hydrostatic pressure suppresses RhoA, reducing MRTF/SRF activity. (A) Normalized RhoA FRET ratio in HT-1080 cells treated with VC or lysophosphatidic acid (LPA; 50 µM; n=40/condition, N=2). $^{**}p < 0.01$. **(B)** Schematic illustrating acute hydrostatic pressure applied to cells. Medium reservoirs were raised to 1 kPa, with one side open and the opposite side sealed with a clip. **(C)** Quantification of RhoA FRET ratio in MDA-MB-231 and HT-1080 cells maintained at 0.08 kPa hydrostatic pressure throughout the experiment. The thick black lines represent the average (n≥50, N=3). $^{*}p < 0.05$. **(D)** Quantification of the nuclear-to-cytoplasmic ratio of MRTF-A-GFP in HT-1080 cells exposed for 10 minutes to 10 %v/v FBS or 10 µM KPT-330 (n=40/condition, N=2). $^{**}p < 0.01$. **(E-F)** Representative confocal images **(E)** and quantification of the nuclear-to-cytoplasmic ratio **(F)** of MRTF-A–GFP in VC- or LPA-treated HT-1080 cells after exposure to 1 kPa for 20 minutes. (n=36/condition, N=3). $^{**,\$,\&,\#}p < 0.01$ (# 1 kPa VC at 0 minutes vs. 20 minutes; & 1 kPa VC at 10 minutes vs. 20 minutes). Scale bar: 35 µm. **(G-H)** Representative confocal images **(G)** and quantification **(H)** of MRTF-A–GFP in HT-1080 cells maintained at 0.08 kPa hydrostatic pressure throughout the experiment. The thick black line represents the average (n=30, N=3). Scale bar: 35 µm. **(I)** Normalized CArG-RE activity in HT-1080 cells treated with VC or CCG-1423 for four days. Dotted line: VC (5 images/experiment, N=3). $^{*}p < 0.05$. Statistics: Unpaired t-test **(A)**, paired t-test **(C)**, Kruskal–Wallis tests followed by Dunn's test **(D)**, two-way ANOVA followed by Tukey's multiple comparison **(F),** Friedman tests followed by Dunn's test **(H)** and unpaired t-test **(I)**. Values: mean ± SD **(A, D)**, mean ± 95% CI **(F)**, symbols & lines **(C, H)**.

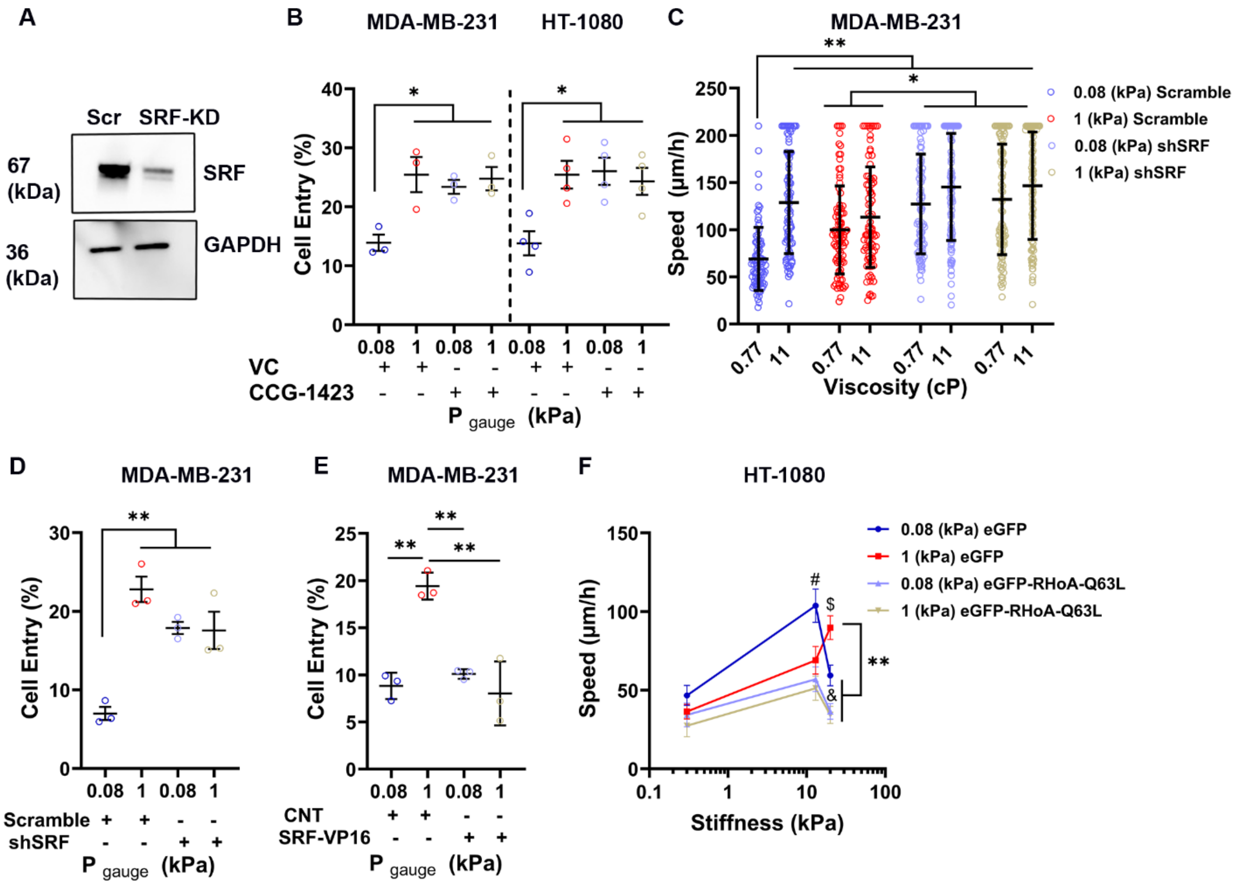

Fig. S6. Rho/MRTF/SRF pathway is a key regulator of cell mechanosensitivity. **(A)** Representative western blot showing the knockdown (KD) efficiency of shSRF in MDA-MB-231 cells (scr: scramble; N=3). **(B)** Percentage of pretreated (0.08 kPa or 1 kPa with VC or CCG-1423) MDA-MB-231 and HT-1080 that enter confined microchannels under flow (ΔP=-0.16 kPa) conditions (>100 cells analyzed/experiment, N≥3). $^*p < 0.05$. **(C)** Speed of pretreated (0.08 kPa or 1 kPa) MDA-MB-231 cells expressing scramble or SRF shRNAs in media of different viscosities (n=90/condition, N=3). $^*p < 0.05$, $^{**}p < 0.01$. **(D)** Percentage of pretreated (0.08 kPa or 1 kPa) MDA-MB-231 cells expressing scramble or SRF shRNAs that enter confined microchannels under flow (ΔP=-0.16 kPa) conditions (>100 cells analyzed/experiment, N=3). $^{**}p < 0.01$. **(E)** Percentage of pretreated (0.08 or 1 kPa) and subsequently transduced (CNT or SRF-VP16) MDA-MB-231 cells that enter confined microchannels under flow (ΔP=-0.16 kPa) conditions (>70 cells analyzed/experiment, N=3). $^{**}p < 0.01$. **(F)** HT-1080 cell speed on substrates of different stiffness after preconditioning at 0.08 or 1 kPa and treating with doxycycline (0.05 μg/mL) to overexpress eGFP or eGFP-RhoA-Q63L. Doxycycline was applied after the pretreatment period for 15 hours (n ≥ 90/condition, N=3). $^{**,\$,\&,\#}p < 0.01$ ($ 1 kPa eGFP on 13 kPa vs. 1 kPa eGFP on 20 kPa; # 0.08 kPa eGFP on 13 kPa vs. 1 kPa eGFP on 13 kPa; & 0.08 kPa eGFP-RhoA-Q63L and 1 kPa eGFP-RhoA-Q63L on 13 kPa vs. same conditions on 20 kPa). Pretreatments **(B-F)**: four days. Statistics: one-way ANOVA followed by Tukey's multiple comparison **(B, D, E)**, two-way ANOVA followed by Tukey's multiple comparison **(C, F)**. Values: mean ± SD **(C)**, mean ± SEM **(B, D, E)**, mean ± 95% CI **(F)**.

Supplementary Video Legends:

Video S1: Migration of pretreated (0.08 kPa) MDA-MB-231 cells through confined microchannels under flow (ΔP=-0.16 kPa) conditions. Scale bar: 80 µm. Arrow indicates flow direction.

Video S2: Migration of pretreated (1 kPa) MDA-MB-231 cells through confined microchannels under flow (ΔP=-0.16 kPa) conditions. Scale bar: 80 µm. Arrow indicates flow direction.

Video S3: Directional choices of pretreated (0.08 kPa) HT-1080 cells in trifurcating, Ψ-like, confined microchannels. Scale bar: 165 µm.

Video S4: Directional choices of pretreated (1 kPa) HT-1080 cells in trifurcating, Ψ-like, confined microchannels. Scale bar: 165 µm.
